# A chiral LC–MS strategy for stereochemical assignment of natural products sharing a 3-methylpent-4-en-2-ol moiety in their terminal structures

**DOI:** 10.3762/bjoc.21.171

**Published:** 2025-10-23

**Authors:** Rei Suo, Raku Irie, Hinako Nakayama, Yuta Ishimaru, Yuya Akama, Masato Oikawa, Shiro Itoi

**Affiliations:** 1 College of Bioresource Sciences, Nihon University, Kameino 1866, Fujisawa, Kanagawa 252-0880, Japan; 2 Yokohama City University, Seto 22-2, Kanazawa-ku, Yokohama, Kanagawa 236-0027, Japan

**Keywords:** chemical degradation, chiral LC–MS analysis, methyl 3-hydroxy-2-methylbutanoate, 3-methylpent-4-en-2-ol moiety, *p*-nitrobenzoyl ester

## Abstract

A terminal 3-methylpent-4-en-2-ol (MPO) moiety is a common structural feature in various polyketide natural products. Stereochemical assignments of this moiety have mainly relied on computational analyses of NMR, ECD, and specific rotation data. However, none of these approaches can be applied to all compounds. In this study, we developed a method to determine the absolute configuration of the terminal MPO moiety with high accuracy and sensitivity by a combination of chemical degradation, chemical synthesis, and chiral LC–MS analysis. The applicability of this method was demonstrated through the stereochemical assignment of (+)-capsulactone (**1**).

## Introduction

Configurational elucidation of natural products is essential for progress in diverse research areas, including the development of synthetic methodologies, the investigation of modes of action, and the evaluation of pharmacological potential, among others. Advancements in spectroscopic techniques, such as nuclear magnetic resonance (NMR) and mass spectrometry (MS), have enabled the structural elucidation of complex natural products at microgram quantities, facilitating the discovery of novel bioactive compounds [1–2]. However, a definitive approach for stereochemical determination that can be applied across all compound classes has yet to be established. Relative configurations are typically determined using NMR-based techniques, such as coupling constant analysis and NOE experiments, sometimes with the aid of computational chemistry [3–7]. In contrast, absolute configuration remains more challenging to determine, as it frequently requires chemical degradation or derivatization. Several derivatization methods using chiral anisotropic reagents, including α-methoxy-α-trifluoromethylphenylacetic acid (MTPA), phenylglycine methyl ester (PGME), and Marfey’s reagents, are widely used [8–10], although their applicability is restricted by the presence of specific functional groups. Total synthesis is a powerful approach for determining absolute configuration through the comparison of specific rotation or chromatographic behavior; however, it requires considerable time and effort. In this context, we have been working on developing effective approaches to determine the absolute configuration of scarce natural products, including heptavalinamide A [11] and poecillastrin C [12–13] by a combination of chemical degradation, chemical synthesis, and liquid chromatography–mass spectrometry (LC–MS) analysis.

**Figure 1:**
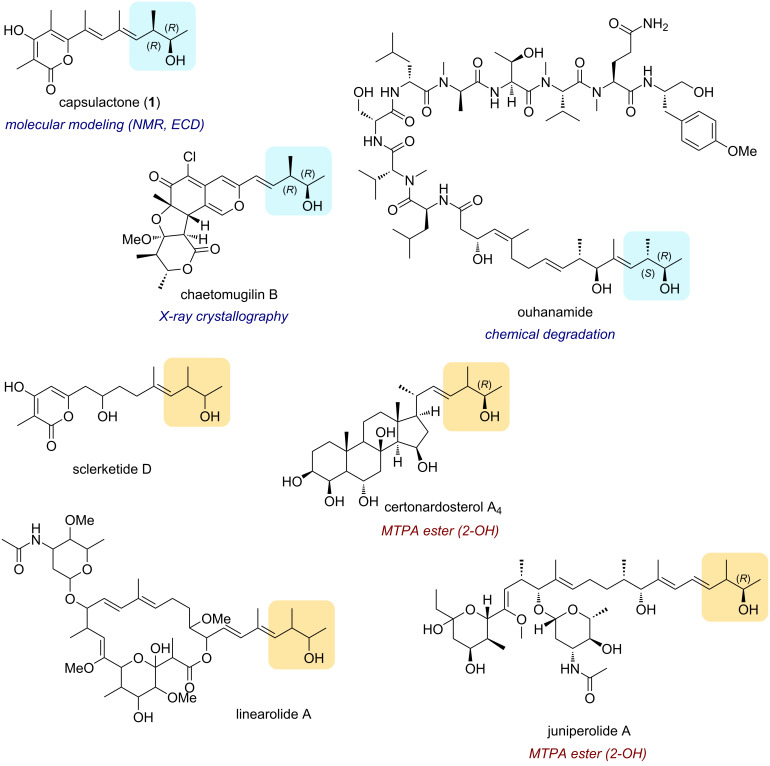
Representative natural products sharing the 3-methylpent-4-en-2-ol (MPO) moiety in their terminal structures, with the moiety highlighted in blue (assigned configuration) or yellow (undetermined configuration). Methods for absolute configuration determination indicated.

The 3-methylpent-4-en-2-ol (MPO) moiety is commonly found at the terminal position of various polyketide natural products such as a series of azaphilones including chaetomugilins [14], chaetoviridins [14–15], and some other α-pyrone polyketides [16–18] (Figure 1). Among the available strategies for elucidating the stereochemistry of MPO, X-ray crystallographic analysis, and computational methods have been widely used. For example, the absolute configuration of chaetomugilin B [19] was determined by X-ray crystallography, while that of capsulactone (**1**) was established through density functional theory (DFT)-based simulations of NMR chemical shifts and electronic circular dichroism (ECD) spectra [17]. However, despite their utility, the general applicability of these computational approaches to MPO-containing molecules is uncertain due to their high conformational flexibility. In other cases, the absolute configurations of compounds such as linearolides [20], juniperolide A [21], certonardosterol A_4_ [22], and sclerketide D [23] remain unresolved due to the limited sample quantity or ambiguous results, even when the modified Mosher’s method is employed; a medium vicinal coupling constant (4–6 Hz) prevents reliable differentiation between *threo* and *erythro* configurations of the adjacent hydroxy group and methyl group (Figure 1) [24]. Therefore, a general and reliable chemical approach for the stereochemical determination of the terminal MPO-containing compounds is required. Herein, we report the development of a method for determining the absolute configuration of the MPO moiety by LC–MS and demonstrate its application to the stereochemical assignment of capsulactone (**1**) at the microgram scale. The strategy involves the optical resolution of MPO derivatives, chemical degradation of **1**, and the stereoselective synthesis of four diastereomers.

## Results and Discussion

**Scheme 1:**
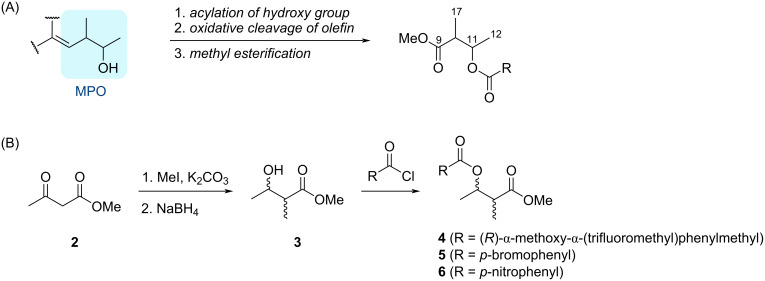
(A) General strategy for the preparation of the fragment from an MPO-containing natural product. (B) Synthesis of esters **4**–**6**, each as a mixture of four stereoisomers.

Our degradation strategy of natural products bearing an MPO moiety includes (1) acylation of hydroxy group, (2) oxidative cleavage of olefin to generate 3-acyloxy-2-methylbutanoic acid, and (3) its methyl esterification (Scheme 1A). We initially investigated derivatization strategies to enable LC–MS detection of the MPO-derived fragment and to achieve a separation of four candidate stereoisomers. To this end, methylation of commercially available methyl acetoacetate (**2**) and subsequent reduction of the ketone carbonyl group was carried out to prepare a mixture of four stereoisomers of methyl 3-hydroxy-2-methylbutanoate (**3**) [25–26]. We then explored LC–MS conditions for their separation. In our initial trial, the stereoisomeric mixture of **3** was successfully separated by preparative high performance liquid chromatography (HPLC) using a chiral column (data not shown). However, each stereoisomer obtained suffered from co-evaporation during solvent removal under reduced pressure, which led us to consider that degradation of the natural product to obtain the corresponding fragment would be challenging. Accordingly, esterification of the hydroxy group at C3 in **3**, and suitable acyl groups were then investigated. First, alcohol **3** was converted to (*R*)-MTPA ester **4** (Scheme 1B). Despite numerous attempts to optimize the chromatographic conditions, the resulting diastereomers could not be separated (Figure S1, Supporting Information File 1). Next, *p*-bromobenzoyl ester **5** was synthesized (Scheme 1B). Although complete separation of the stereoisomers was not achieved, the corresponding peaks exhibited improved resolution compared to those of **4** (Figure S2, Supporting Information File 1). To further enhance separation, the *p*-nitrobenzoyl (PNB) ester **6** was prepared (Scheme 1B). After repeated trials with several chiral HPLC columns and with various elution profiles, the four stereoisomers of **6** were successfully separated on a CHIRALPAK ID-3 column at 40 °C with aqueous MeOH gradient elution (Figure S3, Supporting Information File 1).

**Scheme 2:**
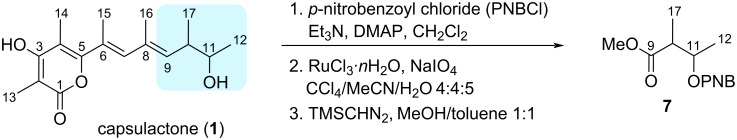
Preparation of the C9–C12 fragment (**7**) from capsulactone (**1**).

**Figure 2:**
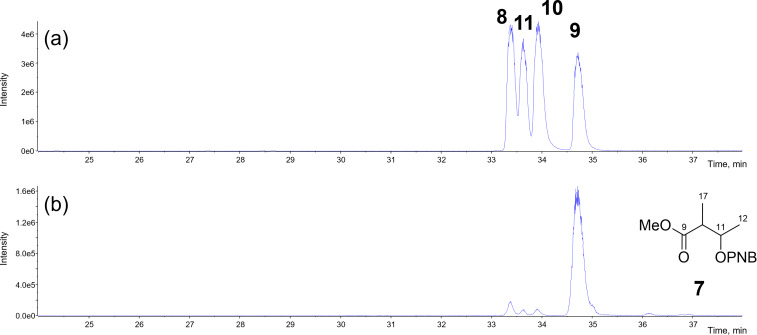
Extracted LC–MS chromatograms (*m*/*z* 304.1) of 4-methoxy-3-methyl-4-oxobutan-2-yl 4-nitrobenzoates. Conditions: CHIRALPAK ID-3 (4.6 × 250 mm, 3 μm) at a flow rate of 0.6 mL/min at 40 °C, with gradient elution from 50% MeOH to 100% MeOH. (a) Synthetic (2*S*,3*S*)-**8**, (2*S*,3*R*)-**9,** (2*R*,3*R*)-**10**, and (2*R*,3*S*)-**11** and (b) C9–C12 fragment **7** derived from **1**.

The developed method for absolute configuration assignment of MPO was applied to the natural product capsulactone (**1**), an α-pyrone polyketide first isolated in 2021 from the endophytic fungus *Penicillium capsulatum* [17], and re-isolated in 2023 from the endophytic fungus *Neurospora dictyophora* WZ-497 [18], whose absolute configuration had been determined by computational methods. We had also independently re-isolated compound **1** (1.2 mg) from the fungus *Fusarium* sp. (see Supporting Information File 1) and subsequently subjected 100 μg of **1** to the sequential degradation steps; (1) *p*-nitrobenzoylation of the two hydroxy groups, (2) RuO_4_ oxidation to cleave olefins including that at C8–C9, and (3) methyl esterification (Scheme 2). The resulting C9–C12 fragment **7** was successfully detected at *m/z* 304.1 [M + Na]^+^ by LC–MS, as expected (see Figure 2b for the chromatogram).

**Scheme 3:**
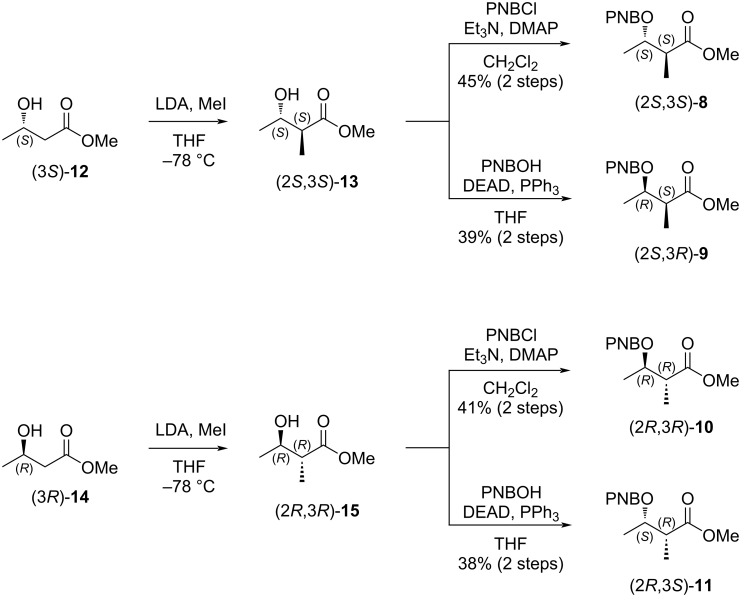
Synthesis of 4-methoxy-3-methyl-4-oxobutan-2-yl 4-nitrobenzoates (2*S*,3*S*)-**8**, (2*S*,3*R*)-**9,** (2*R*,3*R*)-**10**, and (2*R*,3*S*)-**11**.

We then proceeded with the stereoselective synthesis of four diastereomers; 4-methoxy-3-methyl-4-oxobutan-2-yl 4-nitrobenzoates (2*S*,3*S*)-**8**, (2*S*,3*R*)-**9**, (2*R*,3*R*)-**10**, and (2*R*,3*S*)-**11**, as outlined in Scheme 3. The synthesis of (2*S*,3*S*)-**8**, and (2*S*,3*R*)-**9** started from commercially available (3*S*)-**12**, and the stereogenic center at C2 was constructed via stereoselective methylation [27] to afford (2*S*,3*S*)-**13**. Due to the volatility of **13**, the crude solution obtained after the workup (quenching and phase separation) was directly used in the subsequent reaction without purification. Esterification of (2*S*,3*S*)-**13** with *p*-nitrobenzoyl chloride (PNBCl) afforded PNB ester (2*S*,3*S*)-**8** in 45% yield for two steps. Alternatively, PNB ester (2*S*,3*R*)-**9**, the C3-epimer of **8**, was synthesized from (2*S*,3*S*)-**13** via a Mitsunobu reaction using *p*-nitrobenzoic acid (PNBOH) and DEAD in 39% yield for two steps [28]. The stereoisomers (2*R*,3*R*)-**10**, and (2*R*,3*S*)-**11** were prepared in the same manner starting from commercially available (3*R*)-**14**, in acceptable yields of 41% and 38% for two steps, respectively. The four stereoisomers **8**–**11** were separated by LC–MS with the following retention times: (2*S*,3*S*)-**8** (33.4 min), (2*S*,3*R*)-**9** (33.5 min)**,** (2*R*,3*R*)-**10** (33.9 min), (2*R*,3*S*)-**11** (34.7 min) (Figure 2a). The LC–MS analysis of fragment **7**, derived from natural **1**, showed a retention time identical to that of (2*R*,3*S*)-**11** (Figure 2b), thus assigning the absolute configuration of the MPO moiety in **1** as (10*R*,11*R*) [29]. This assignment was consistent with prior reports, and the observed specific rotation of **1**, measured as [α]_D_^26^ +12 (*c* 0.1, MeOH) closely matched the reported values; [α]_D_^21^ +12 (*c* 0.1, MeOH) [17], and [α]_D_^20^ +12.8 (*c* 0.24, MeOH) [18].

Shortly before our report, the absolute configuration of the MPO moiety in ouhanamide [30] (Figure 1), isolated from a cyanobacterium, was determined using a similar approach involving *p*-bromobenzoylation followed by ozonolysis to generate 3-((4-bromobenzoyl)oxy)-2-methylbutanoic acid. The four stereoisomers of this fragment were prepared through two separate Evans aldol reactions, each affording a pair of diastereomers. The final four stereoisomers were resolved by chiral HPLC (CHIRALPAK IA column with hexane/EtOH/TFA isocratic elution). While this strategy provided a reliable assignment, it required multiple synthetic steps, including installation and removal of chiral auxiliaries, as well as extensive chromatographic operations. Moreover, the use of TFA in the mobile phase rendered the method incompatible with LC–MS detection, necessitating a relatively larger amount of sample for analysis. In contrast, a key advantage of our method is the use of a PNB group as the acyl moiety, which enables direct conversion to a C3-epimer via a Mitsunobu reaction, thereby streamlining the synthesis of the four stereoisomers. Additionally, the reversed-phase HPLC conditions without TFA in the mobile phase are compatible with LC–MS, enabling highly sensitive detection, and facilitating reliable stereochemical assignment using only a minimal amount (less than a microgram) of a natural product derivative.

## Conclusion

In conclusion, we have developed a method for the stereochemical determination of the terminal MPO moiety with high accuracy and sensitivity, based on a combination of chemical degradation, efficient synthesis of all stereoisomers of the fragment, and LC–MS analysis using a chiral stationary phase. The applicability of this method was validated through the successful determination of the absolute configuration of capsulactone (**1**) using only 100 μg of sample. This is the first report to establish the elution pattern of the four diastereomers of 3-hydroxy-2-methylbutanoic acid derivatives by LC–MS. Notably, the method developed herein enables the assignment of absolute configuration without the need for the individual synthesis of all four stereoisomers, as the use of stereomixture **6** is sufficient for the stereochemical assignment. This represents a significant advantage over previously reported chemical methods [24,30]. Our method would be applicable to a variety of natural products, such as linearomide, juniperolide A, certonardosterol A_4_, and sclerketide D, all of which share an undetermined MPO moiety in their terminal structures. Given the utility of this strategy, it holds promise as a valuable tool for the structural determination of diverse MPO-containing natural products in future studies.

## Supporting Information

File 1 Experimental section, LC–MS chromatograms and NMR spectra.

## Data Availability

All data that supports the findings of this study is available in the published article and/or the supporting information of this article.

## References

[1] Molinski T F, Morinaka B I (2012). Tetrahedron.

[2] Menna M, Imperatore C, Mangoni A, Della Sala G, Taglialatela-Scafati O (2019). Nat Prod Rep.

[3] Matsumori N, Kaneno D, Murata M, Nakamura H, Tachibana K (1999). J Org Chem.

[4] Higashibayashi S, Czechtizky W, Kobayashi Y, Kishi Y (2003). J Am Chem Soc.

[5] Petrovic A G, Navarro-Vazquez A, Lorenzo Alonso-Gomez J (2010). Curr Org Chem.

[6] Bertucci C, Tedesco D (2012). J Chromatogr A.

[7] Harada N, Nakanishi K (1972). Acc Chem Res.

[8] Dale J A, Mosher H S (1973). J Am Chem Soc.

[9] Kusumi T, Takahashi H, Xu P, Fukushima T, Asakawa Y, Hashimoto T, Kan Y, Inouye Y (1994). Tetrahedron Lett.

[10] Marfey P (1984). Carlsberg Res Commun.

[11] Suo R, Watanabe R, Takada K, Suzuki T, Oikawa H, Itoi S, Sugita H, Matsunaga S (2020). Org Lett.

[12] Irie R, Takada K, Ise Y, Ohtsuka S, Okada S, Gustafson K R, Matsunaga S (2017). Org Lett.

[13] Irie R, Hitora Y, Watanabe R, Clark H, Suyama Y, Sekiya S, Suzuki T, Takada K, Matsunaga S, Hosokawa S (2024). Org Lett.

[14] Gao J-M, Yang S-X, Qin J-C (2013). Chem Rev.

[15] Chen C, Tao H, Chen W, Yang B, Zhou X, Luo X, Liu Y (2020). RSC Adv.

[16] Ma Y, Wen Y, Cheng H, Deng J, Peng Y, Bahetejiang Y, Huang H, Wu C, Yang X, Pang K (2021). Bioorg Med Chem Lett.

[17] Xian P-J, Chen H-Y, Feng Z, Zhao W, Yang X-L (2021). J Asian Nat Prod Res.

[18] Li X, Gong Y-X, Feng L, Wang X-J, Wang J-W, Zhang A-X, Tan N-H, Wang Z (2023). Phytochemistry.

[19] Yamada T, Doi M, Shigeta H, Muroga Y, Hosoe S, Numata A, Tanaka R (2008). Tetrahedron Lett.

[20] Ueki M, Koshiro N, Aono H, Kawatani M, Uramoto M, Kawasaki H, Osada H (2013). J Antibiot.

[21] Raju R, Gromyko O, Fedorenko V, Luzhetskyy A, Plaza A, Müller R (2012). Org Lett.

[22] Wang W, Jang H, Hong J, Lee C-O, Im K S, Bae S-J, Jung J H (2004). J Nat Prod.

[23] Liu Z, Qiu P, Liu H, Li J, Shao C, Yan T, Cao W, She Z (2019). Bioorg Chem.

[24] Neuhaus G F, Adpressa D A, Bruhn T, Loesgen S (2019). J Nat Prod.

[25] Trotta A H (2015). Org Lett.

[26] Kalaitzakis D, Kambourakis S, Rozzell D J, Smonou I (2007). Tetrahedron: Asymmetry.

[27] Jacolot M, Jean M, Levoin N, van de Weghe P (2012). Org Lett.

[28] Mitsunobu O (1981). Synthesis.

[R29] 29Note that Cahn–Ingold–Prelog priorities at C2 in **8**–**11** are reversed from that at C10 in **1**; i.e., if the configuration in the degradation fragment **7** is found to be (10*S*), then the absolute configuration at C10 in **1** is (10*R*).

[30] Niiyama M, Kurisawa N, Umeda K, Jeelani G, Agusta A L, Nozaki T, Suenaga K (2025). J Nat Prod.

