# Supplementary material for: A chiral LC–MS strategy for stereochemical assignment of natural products sharing a 3-methylpent-4-en-2-ol moiety in their terminal structures
Source: Beilstein J Org Chem. 2025 Oct 23;21:2243–9. doi: 10.3762/bjoc.21.171 (PMC12557429; doi:10.3762/bjoc.21.171)
Supplement: File 1 — Experimental section, LC–MS chromatograms and NMR spectra. [file Beilstein_J_Org_Chem-21-2243-s001.pdf]

BEILSTEIN JOURNAL OF ORGANIC CHEMISTRY

Supporting Information

for

A chiral LC–MS strategy for stereochemical assignment of natural products sharing a 3-methylpent-4-en-2-ol moiety in their terminal structures

Rei Suo, Raku Irie, Hinako Nakayama, Yuta Ishimaru, Yuya Akama, Masato Oikawa and Shiro Itoi

Beilstein J. Org. Chem. **2025,** *21,* 2243–2249. doi:10.3762/bjoc.21.171

Experimental section, LC–MS chromatograms and NMR spectra

License and Terms: This is a supporting information file under the terms of the Creative Commons Attribution License (https://creativecommons.org/licenses/by/4.0). Please note that the reuse, redistribution and reproduction in particular requires that the author(s) and source are credited and that individual graphics may be subject to special legal provisions.
The license is subject to the *Beilstein Journal of Organic Chemistry* terms and conditions: (https://www.beilstein-journals.org/bjoc/terms)

Table of contents

1. Experimental section S2–S8

General experimental procedures

Fermentation, extraction and isolation of capsulactone (**1**)

Synthetic procedures for esters **4**–**6**, each as a mixture of four stereoisomers

Synthetic procedures for PNB esters **8**–**11**

Degradation of capsulactone (**1**) to detect the C9–C12 fragment

2. LC–MS chromatograms S9–S13

Figure S1. LC–MS chromatograms of esters **4**–**6** using CHIRALPAK® IC (MeOH conditions)

Figure S2. LC–MS chromatograms of esters **4**–**6** using CHIRALPAK® IF (MeOH conditions)

Figure S3. LC–MS chromatograms of esters **4**–**6** using CHIRALPAK® ID-3 (MeOH conditions)

Table S1. Enantiomeric and diastereomeric composition of synthetic standards **8**–**11** determined by chiral HPLC

Figure S4. HPLC chromatograms of **8**–**11** using CHIRALPAK® ID-3

3. NMR spectra S13–S21

Table S2. ^{1}H NMR data (500 MHz) of capsulactone (**1**) in CD_3OD

Figure S5–S19 NMR spectra of capsulactone (**1**) and compounds **S1**, **3**, **8**, **9**, **10** and **11**

1. Experimental section

General experimental procedures

Optical rotations were measured on a JASCO P-2300 polarimeter. UV spectra were measured on a JASCO V-730 spectrophotometer. IR spectra were recorded on a JASCO FT/IR-400 spectrometer. NMR spectra were measured on a JEOL alpha 500 spectrometer or a Bruker AVANCE III HD 400 spectrometer using residual solvent signals (δ_H 3.30; δ_C 49.0 ppm of CD_3OD and δ_H 7.24; δ_C 77.0 ppm of $CDCl_3$) as internal standards. LC–MS experiments were performed on a Shimadzu LC-20AD solvent delivery system interfaced with a SCIEX X500R Q-TOF mass spectrometer (ESI source), or a Thermo Scientific UltiMate 3000 basic automated system interfaced with a Thermo Fisher Scientific Q Exactive Focus mass spectrometer (ESI source). Analytical or preparative thin-layer chromatography (TLC) was performed using a Merck silica gel 60 F254 plate (0.25 or 0.50 mm thickness). Flash column chromatography was carried out using Kanto chemical silica gel 60N (40–50 mesh) or Yamazen silica gel HiFlash (SiOH-30 μ Premium, 30 μm, 60 Å) with an automated flash column system EPCLC-Wprep2XY-10VW (Yamazen Corporation). All reactions susceptible to moisture and air were carried out in an atmosphere of argon gas, using glassware oven-dried over 3 h. CH_2Cl_2 and THF were purified by a Glass Contour Solvent Dispensing System (Nikko Hansen). All other reagents were purchased at the highest commercial grade and used directly.

Fermentation, extraction and isolation of capsulactone (1)

Fusarium sp. was grown on rice solid medium (to 70 g of Japanese commercially available rice was added 70 mL of sterile sea water and 70 mL of distilled water) at room temperature. After 25 days, the whole culture was extracted with EtOAc (300 mL). The extracts were combined, concentrated in vacuo, and partitioned between EtOAc and H_2O. The dried EtOAc extract was subjected to ODS flash column chromatography with stepwise elution of 5% MeOH, 50% MeOH, 90% MeOH, 100% MeOH and $CHCl_3$/MeOH 1:1. The 90% MeOH fraction was first purified by RP-HPLC on a Cosmosil 5C$_{18}$-AR-II column (10 × 250 mm) with gradient elution from 60% MeOH to 90% MeOH over 30 min in the presence of 0.5% AcOH at a flow rate of 4 mL/min to afford several fractions. Each fraction was further purified by RP-HPLC on a Cosmosil 5C$_{18}$-AR-II column (4.6 × 250 mm) with gradient elution from 36% MeCN to 52% MeCN over 30 min in the presence of 0.5% AcOH at a flow rate of 1mL/min to give capsulactone (**1**, 1.2 mg).

Capsulactone (**1**); colorless gum; $[\alpha]_D^{26}$ +12 (*c* 0.1, MeOH); UV (MeOH) λ_{max} (log ε) 308 (3.6), 250 (3.6); ^{1}H and ^{13}C NMR data, see Table 1; HRMS (ESI, positive) calcd for $C_{17}H_{24}O_4$ [(M+H)$^+$] 293.1747, found 293.1741.

Synthetic procedures for esters 4–6, each as a mixture of four stereoisomers

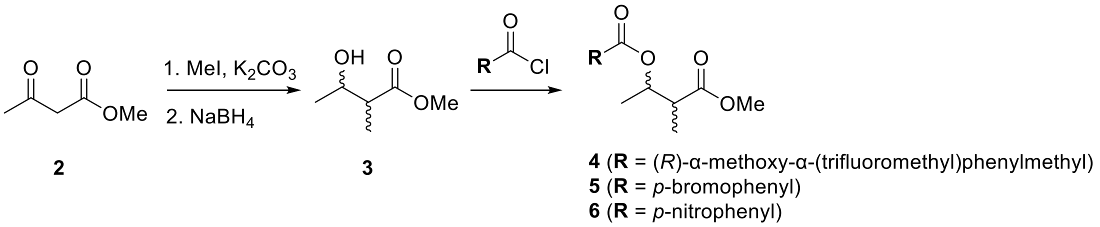

Methyl 2-methyl-3-oxobutanoate (S1), and methyl-3-hydroxy-2-methylbutanoate (3)

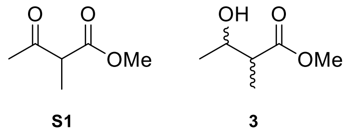

To a stirred mixture of methyl acetoacetate (**2**, 0.500 mL, 4.65 mmol) and iodomethane (0.290 mL, 4.65 mmol) at 0 °C was added K_2CO_3 (965.2 mg, 6.98 mmol) portionwise over 5 min. After stirring at 0 °C for 1.5 h, the mixture was allowed to warm to rt. After 19 h, to the mixture was added Et_2O (5 mL), and the resulting suspension was filtered. The filtrate was washed with brine (5 mL), dried over Na_2SO_4, and concentrated in vacuo. The residue was purified by silica gel column chromatography (SiOH-30μ Premium, 14 g, EtOAc/hexane 1:4) to give racemic **S1** (377.3 mg, 62%) as a colorless syrup: ^{1}H NMR (selected for the keto tautomer, 400 MHz, $CDCl_3$) δ 3.72 (s, 3H), 3.50 (q, *J* = 7.2 Hz, 1H), 2.22 (s, 3H), 1.33 (d, *J* = 7.1 Hz, 3H). Other spectroscopic data for **S1** were in good agreement with those reported.[1]

To a stirred suspension of $NaBH_4$ (9.3 mg, 0.25 mmol) in EtOH (3.0 mL) at 0 °C was added a solution of ketone **S1** (100.2 mg, 0.770 mmol), thus obtained above, in EtOH (2.0 mL) in a dropwise manner over 3 min. After stirring at 0 °C for 1.5 h, to the mixture was added saturated aqueous NH_4Cl (0.5 mL). The mixture was partitioned between EtOAc and H_2O (5 mL each). The aqueous layer was separated and extracted with EtOAc (2 × 5 mL). Combined extracts were washed with brine (5 mL), dried over Na_2SO_4, and concentrated in vacuo. The residue was purified by silica gel column chromatography (SiOH-30μ Premium, 7 g, EtOAc/hexane 2:3) to give alcohol **3** (a mixture of four stereoisomers, 65.3 mg, 64%) as a colorless syrup: ^{1}H NMR (400 MHz, $CDCl_3$) δ 4.05 and 3.86 (two multiplets, 1H total), 3.70 and 3.69 (two singlets, 3H total), 2.59 and 2.49 (two multiplets, 1H total), 2.49 and 2.44 (multiplet and double quartet, *J* = 7.2, 7.2 Hz, 1H total), 1.20 and 1,16 (two doublets, *J* = 6.4 Hz each, 3H total), 1.17 and 1.17 (two doublets, *J* = 7.2 Hz each, 3H total). Other spectroscopic data for **3** were in good agreement with those reported.[2]

Methyl 2-methyl-3-(((*R*)-3,3,3-trifluoro-2-methoxy-2-phenylpropanoyl)oxy)butanoate (4)

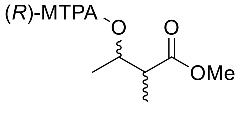

4

To a stirred solution of alcohol **3** (5.0 mg, 0.038 mmol) in pyridine (150 µL) at rt was added (*S*)-(+)-α-methoxy-α-(trifluoromethyl)phenylacetyl chloride ((*S*)-(+)-MTPACl, 10 µL, 0.053 mmol). After 16 h, the reaction mixture was diluted with water (0.7 mL) and extracted with $CHCl_3$ (2 × 0.5 mL). The combined extracts were concentrated in vacuo to afford crude (*R*)-MTPA ester **4** (9.2 mg) as a colorless solid: HRMS (ESI, positive) calcd for $C_{16}H_{19}F_3NaO_5$ [$(M+Na)^+$] 371.1077, found 371.1082.

4-Methoxy-3-methyl-4-oxobutan-2-yl 4-bromobenzoate (5)

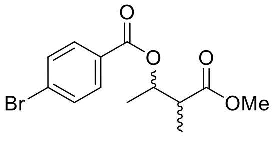

5

To a stirred solution of alcohol **3** (5.0 mg, 0.038 mmol) in pyridine (180 µL) at rt were added *p*-bromobenzoyl chloride (20.9 mg, 0.0952 mmol) and DMAP (1.7 mg, 0.014 mmol). After 16 h, the reaction mixture was diluted with water (0.8 mL) and extracted with $CHCl_3$ (2 × 0.5 mL). The combined extracts were concentrated in vacuo to afford crude ester **5** (12.9 mg) as a white solid: HRMS (ESI, positive) calcd for $C_{13}H_{15}BrNaO_4$ [$(M+Na)^+$] 337.0046, found 337.0058.

4-Methoxy-3-methyl-4-oxobutan-2-yl 4-nitrobenzoate (6)

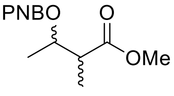

6

To a stirred solution of alcohol **3** (5.0 mg, 0.038 mmol) in CH_2Cl_2 (300 µL) at rt were added Et_3N (21 µL, 0.151 mmol), DMAP (1.45 mg, 0.012 mmol) and *p*-nitrobenzoyl chloride (21.6 mg, 0.117 mmol). After 5 h, the reaction mixture was concentrated in vacuo to afford crude ester **6** (14.9 mg) as a yellow solid: HRMS (ESI, positive) calcd for $C_{13}H_{15}NNaO_6$ [$(M+Na)^+$] 304.0792, found 304.0796.

Synthetic procedures for PNB esters 8–11

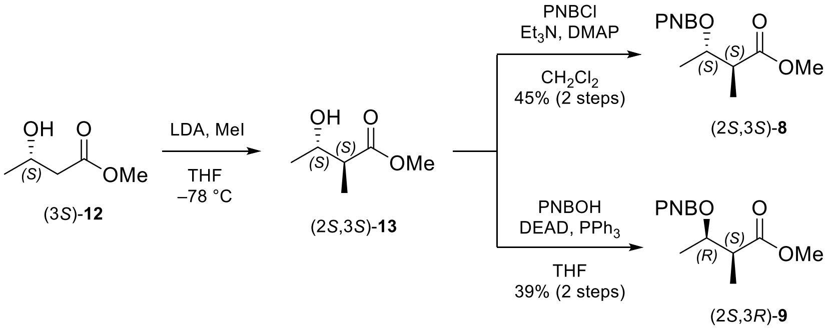

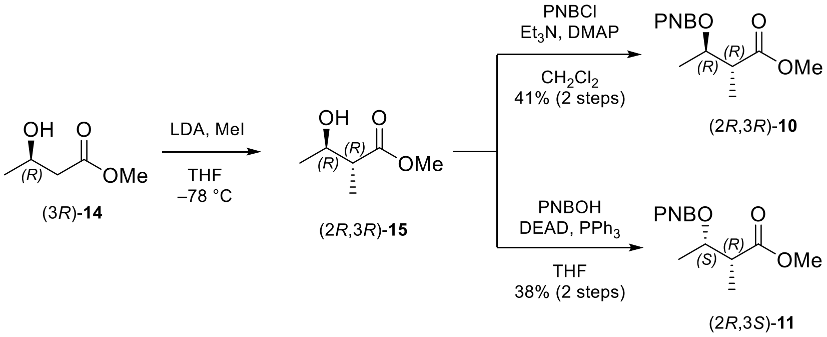

Methyl (2*S*,3*S*)-3-hydroxy-2-methylbutanoate (13), and (2*S*,3*S*)-4-methoxy-3-methyl-4-oxobutan-2-yl 4-nitrobenzoate (8)

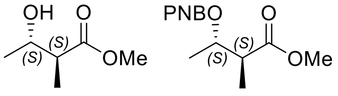

(2*S*,3*S*)-**13** (2*S*,3*S*)-**8**

To a stirred solution of LDA (1.0 M in THF/hexanes, 25 mL, 25 mmol) at −78 °C was added methyl (*S*)-(+)-3-hydroxybutyrate (**12**, 0.928 mL, 8.33 mmol) in THF (8.0 mL) in a dropwise manner for 3 min. After stirring at −78 °C for 30 min, to the solution was added iodomethane (3.11 mL, 49.5 mmol) in a dropwise manner over 3 min. After stirring at −78 °C for additional 2 h, the mixture was then allowed to warm to 0 °C and quenched with hydrochloric acid (1 M, 32 mL). The aqueous layer was separated and extracted with CH_2Cl_2 (3 × 30 mL). Combined extracts were washed with brine (2 × 60 mL), dried over Na_2SO_4, and concentrated in vacuo to a volume of ca. 5 mL to afford a yellow solution of crude **13** (6.54 g), which was used for the next reaction without purification.

A part of the residual solution of crude **13** (129.8 mg), thus obtained above, was diluted with CH_2Cl_2 (1.0 mL). To the stirred solution at rt were added Et_3N (69.0 μL, 0.495 mmol), DMAP (2.7 mg, 0.022 mmol) and *p*-nitrobenzoyl chloride (PNBCl, 46.3 mg, 0.250 mmol). After 1 h, the mixture was poured into saturated aqueous NH_4Cl (1 mL). The aqueous layer was separated and extracted with CH_2Cl_2 (3 × 1 mL). The combined extracts were washed with brine (2 × 1 mL), dried over Na_2SO_4, and concentrated in vacuo. The residue was purified by preparative TLC (0.50 mm thickness, 20 × 20 cm, EtOAc/hexane 1:4) to give PNB ester **8** (21.2 mg, 45% for 2 steps) as a colorless oil: $[\alpha]_D^{26}$ +49 (*c* 1.0, MeOH); IR (ATR) 2987, 2952, 1726, 1726, 1528, 1274, 720 cm^{-1}; ^1H NMR (400 MHz, $CDCl_3$) δ 8.26 (m, 2H), 8.15 (m, 2H), 5.35 (dq, *J* = 6.5, 6.5 Hz, 1H), 3.65 (s, 3H), 2.86 (dq, *J* = 7.2,

7.2 Hz, 1H), 1.37 (d, J = 6.4 Hz, 3H), 1.23 (d, J = 7.2 Hz, 3H); ^{13}C NMR (100 MHz, $CDCl_3$) δ 173.8, 163.7, 150.6, 135.7, 130.7 (×2), 123.5 (×2), 73.4, 51.9, 44.6, 17.1, 12.8; HRMS (ESI, positive) calcd for $C_{13}H_{15}NNaO_6$ [$(M+Na)^+$] 304.0792, found 304.0796.

(2*S*,3*R*)-4-Methoxy-3-methyl-4-oxobutan-2-yl 4-nitrobenzoate (9)[3]

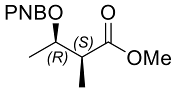

(2*S*,3*R*)-**9**

A part of the residual solution of crude **13** (125.1 mg), thus obtained above, was diluted with THF (1.0 mL). To the stirred solution at 0 °C were added *p*-nitrobenzoic acid (PNBOH, 54.6 mg, 0.327 mmol), PPh_3 (86.5 mg, 0.330 mmol), and a solution of diethyl azodicarboxylate in toluene (2.2 M, 0.148 mL, 0.326 mmol). After stirring at rt for 2 h, the mixture was concentrated in vacuo. The residue was purified by silica gel column chromatography (SiOH-30μ Premium, 7 g, EtOAc/hexane 23:77) to give PNB ester **9** (18.3 mg, 39% for 2 steps) as a colorless oil: $[\alpha]_D^{26}$ –14 (*c* 1.0, MeOH); IR (ATR) 2990, 2952, 1728, 1728, 1529, 1275, 720 cm^{-1}; ^{1}H NMR (400 MHz, $CDCl_3$) δ 8.26 (m, 2H), 8.16 (m, 2H), 5.43 (m, 1H), 3.67 (s, 3H), 2.79 (qd, J = 7.1, 5.5 Hz, 1H), 1.39 (d, J = 6.4 Hz, 3H), 1.27 (d, J = 7.1 Hz, 3H); ^{13}C NMR (100 MHz, $CDCl_3$) δ 173.7, 163.9, 150.6, 135.7, 130.7, 123.5, 72.8, 51.9, 44.3, 17.5, 12.2; HRMS (ESI, positive) calcd for $C_{13}H_{15}NNaO_6$ [$(M+Na)^+$] 304.0792, found 304.0795.

Methyl (2*R*,3*R*)-3-hydroxy-2-methylbutanoate (15), and
(2*R*,3*R*)-4-methoxy-3-methyl-4-oxobutan-2-yl 4-nitrobenzoate (10)

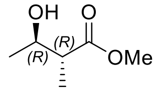

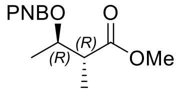

(2*R*,3*R*)-**15** (2*R*,3*R*)-**10**

To a stirred solution of LDA (1.0 M in THF/hexanes, 25 mL, 25 mmol) at −78 °C was added methyl (*R*)-(−)-3-hydroxybutyrate (**14**, 0.928 mL, 8.33 mmol) in THF (8 mL) in a dropwise manner for 3 min. After stirring at −78 °C for 30 min, to the solution was added iodomethane (3.11 mL, 49.5 mmol) in a dropwise manner for 3 min. After stirring at −78 °C for additional 2 h, the mixture was then allowed to warm to 0 °C, poured into hydrochloric acid (1 M, 30 mL), and extracted with CH_2Cl_2 (60 mL, 20 mL, 100 mL). The combined extracts were washed with brine (70 mL), dried over Na_2SO_4, and concentrated in vacuo to a volume of ca. 5 mL to afford a yellow solution of crude **15** (6.36 g), which was used for the next reaction without purification.

A part of the residual solution of crude alcohol **15** (121.5 mg), thus obtained above, was diluted with CH_2Cl_2 (1.0 mL). To the stirred solution at rt were added Et_3N (69.7 μL, 0.500 mmol), DMAP (2.6 mg, 0.021 mmol), and *p*-nitrobenzoyl chloride (PNBCl, 50.0 mg, 0.269 mmol). After 1 h, the mixture was poured into saturated aqueous NH_4Cl (1 mL). The aqueous layer was separated and extracted with CH_2Cl_2 (3 × 1 mL). The combined extracts were washed with brine (2 × 1 mL), dried over Na_2SO_4, and concentrated in vacuo. The residue was purified by preparative TLC (0.50 mm

thickness, 20 × 20 cm, EtOAc/hexane 1:4) to give PNB ester **10** (19.0 mg, 41% for 2 steps) as a colorless oil: $[\alpha]_D^{26}$ −49 (*c* 1.0, MeOH); IR (ATR) 2987, 2952, 1726, 1726, 1528, 1273, 720 cm^{-1}; ^1H NMR (400 MHz, $CDCl_3$) δ 8.25 (m, 2H), 8.15 (m, 2H), 5.35 (m, 1H), 3.65 (s, 3H), 2.85 (dq, *J* = 7.1, 7.1 Hz, 1H), 1.37 (d, *J* = 6.4 Hz, 3H), 1.23 (d, *J* = 7.2 Hz, 3H); ^{13}C NMR (100 MHz, $CDCl_3$) δ 173.8, 163.7, 150.5, 135.7, 130.7, 123.5, 73.4, 51.9, 44.6, 17.1, 12.8; HRMS (ESI, positive) calcd for $C_{13}H_{15}NNaO_6$ [$(M+Na)^+$] 304.0792, found 304.0797.

(2*R*,3*S*)-4-Methoxy-3-methyl-4-oxobutan-2-yl 4-nitrobenzoate (11) [4]

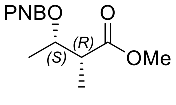

(2*R*,3*S*)-**11**

A part of the residual solution of crude alcohol **15** (113.1 mg), obtained as described above, was diluted with THF (1.0 mL). To the stirred solution at 0 °C were added *p*-nitrobenzoic acid (PNBOH, 56.7 mg, 0.339 mmol), PPh_3 (88.9 mg, 0.339 mmol), and a solution of diethyl azodicarboxylate in toluene (2.2 M, 154 μL, 0.339 mmol). After stirring at rt for 1.5 h, the mixture was concentrated in vacuo. The residue was purified by silica gel column chromatography (60N, 6.9 g, EtOAc/hexane 3:17) to give PNB ester **11** (18.3 mg, 39% for 2 steps) as a colorless oil: $[\alpha]_D^{26}$ +14 (*c* 1.0, MeOH); IR (ATR) 2989, 2951, 1725, 1725, 1528, 1275, 720 cm^{-1}; ^1H NMR (400 MHz, $CDCl_3$) δ 8.26 (m, 2H), 8.16 (m, 2H), 5.43 (m, 1H), 3.67 (s, 3H), 2.79 (qd, *J* = 7.1, 5.5 Hz, 1H), 1.39 (d, *J* = 6.4 Hz, 3H), 1.27 (d, *J* = 7.1 Hz, 3H); ^{13}C NMR (100 MHz, $CDCl_3$) δ 173.7, 163.9, 150.6, 135.7, 130.7, 123.5, 72.8, 51.9, 44.3, 17.5, 12.2; HRMS (ESI, positive) calcd for $C_{13}H_{15}NNaO_6$ [$(M+Na)^+$] 304.0792, found 304.0795.

Degradation of capsulactone (1) to detect the C9–C12 fragment (7)

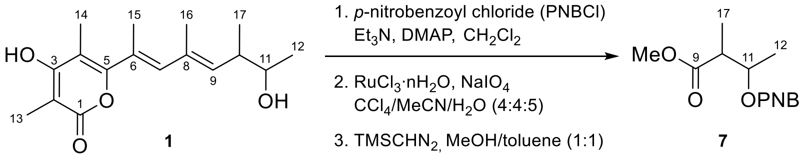

To a solution of capsulactone (**1**, 100 μg, 0.342 μmol) in CH_2Cl_2 (100 μL) at rt were added Et_3N (10.0 μL, 0.072 mmol), *p*-nitrobenzoyl chloride (PNBCl, 2.0 mg, 0.072 mmol) and DMAP (2.0 mg, 0.016 mmol). After stirring overnight, the mixture was concentrated in vacuo to afford crude 4-nitrobenzoyl ester, which was used in the next reaction without purification: ESIMS *m/z* 464.2 [$(M+Na)^+$], *m/z* 613.2 [$(M+Na)^+$].

To a solution of crude nitrobenzoyl ester, thus obtained above, in CCl_4/MeCN/H_2O 4:4:5 (104 μL) at rt were added aqueous $RuCl_3 \cdot nH_2O$ (25 mM, 10 μL, 0.25 μmol) and $NaIO_4$ (2.0 mg, 0.0093 mmol). After 1 h, the mixture was diluted with hydrochloric acid (0.5 M, 100 μL) and extracted with EtOAc (2 × 0.5 mL). The combined extracts were concentrated in vacuo to give crude carboxylic acid, which was used for the next reaction without purification.

To a solution of crude carboxylic acid, obtained as described above, in MeOH and toluene 1:1 (2.0 mL) at rt was added a solution of $TMSCHN_2$ (1.0 M in hexane) until the solution turned yellow. After stirring for 30 min, a few drops of AcOH were added until the yellow color disappeared and then the mixture was concentrated in vacuo to afford crude C9–C12 fragment **7**: HRMS (ESI, positive) calcd for $C_{13}H_{15}NNaO_6$ $[(M+Na)^+]$ 304.0792, found 304.0784.

References

(1) Trotta, A. H. *Org. Lett.* **2015**, *17*, 3358–3361.

(2) Kalaitzakis, D.; Smonou, I. A. *J. Org. Chem.* **2008**, *73*, 3919–3921.

(3) Chang, S.; Hur, S.; Britton, R. *Chem. Eur. J*. **2015**, *21*, 16646–16653.

(4) Zhang, Y.-H.; Song, K.; Zhu, N.-Y.; Yang, D. *Chem. Eur. J*. **2010**, *16*, 577-587.

2. LC–MS chromatograms

LC–MS separation of esters 4–6

Aliquots (1 μL) of each sample were injected into a CHIRALPAK IC (4.6 × 250 mm, 5 μm), CHIRALPAK IF (4.6 × 250 mm, 5 μm), and CHIRALPAK ID-3 (4.6 × 250 mm, 3 μm) at a flow rate of 0.6 mL/min at 40 °C, with gradient elution from 50% MeOH to 100% MeOH, respectively. Gradient elution was performed using solvent A (H_2O) and solvent B (MeOH) with the following linear gradient combination: 50% (B) kept for 3 min, increased to 100% (B) over 20 min, and kept for 15 min. Gradient elution was also performed using MeCN, but each combination was unsuccessful. The LC–MS chromatograms performed using CHIRALPAK IC, IF, ID-3 in MeOH condition were shown as below.

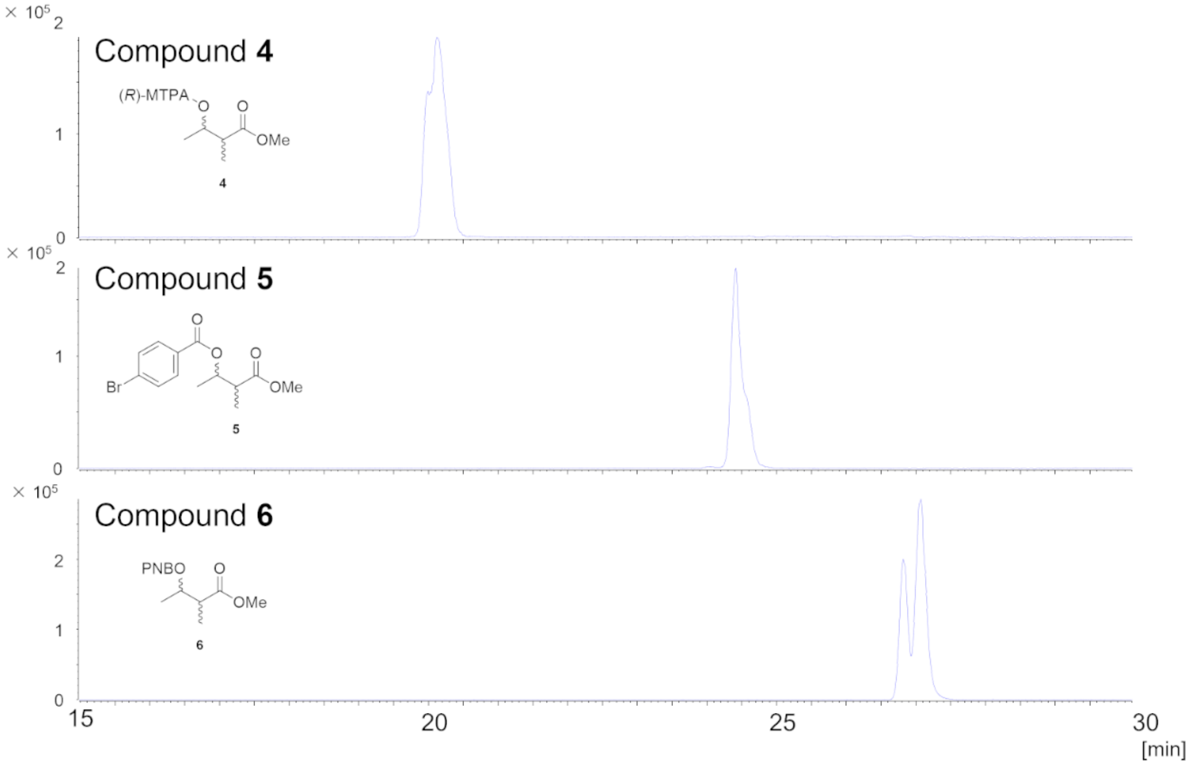

Figure S1. LC–MS chromatograms of esters **4**–**6** using CHIRALPAK IC (MeOH conditions).

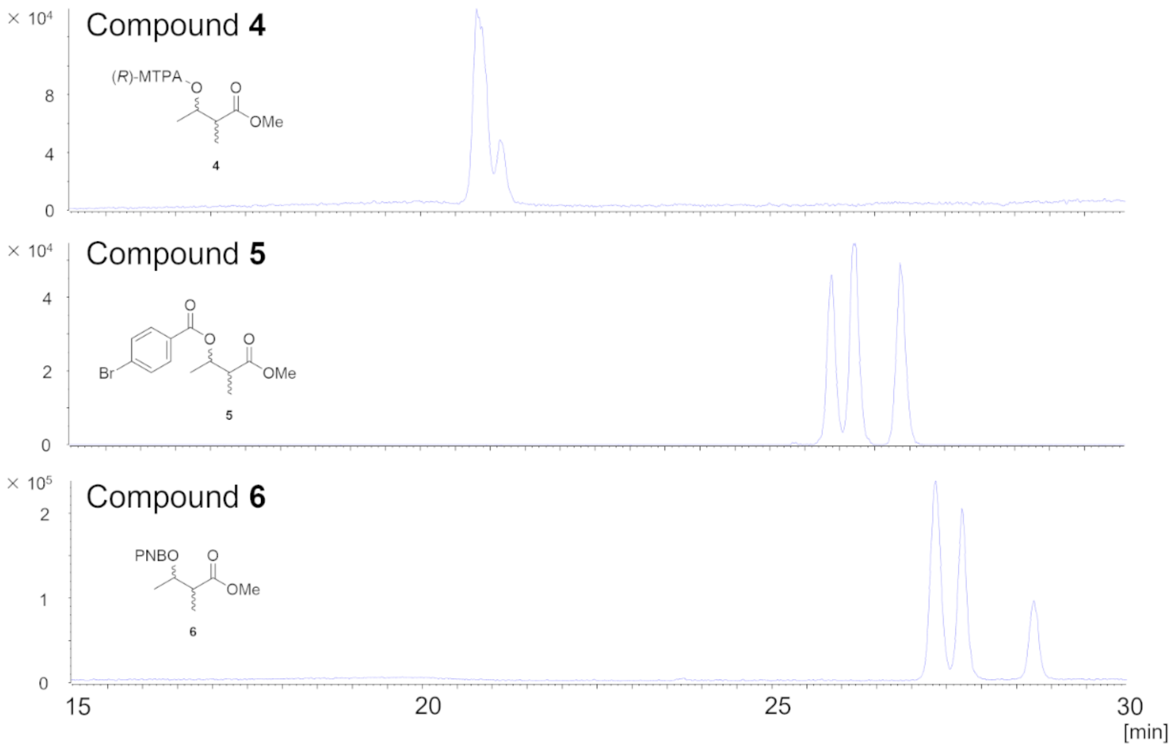

Figure S2. LC–MS chromatograms of esters **4**–**6** using CHIRALPAK IF (MeOH conditions).

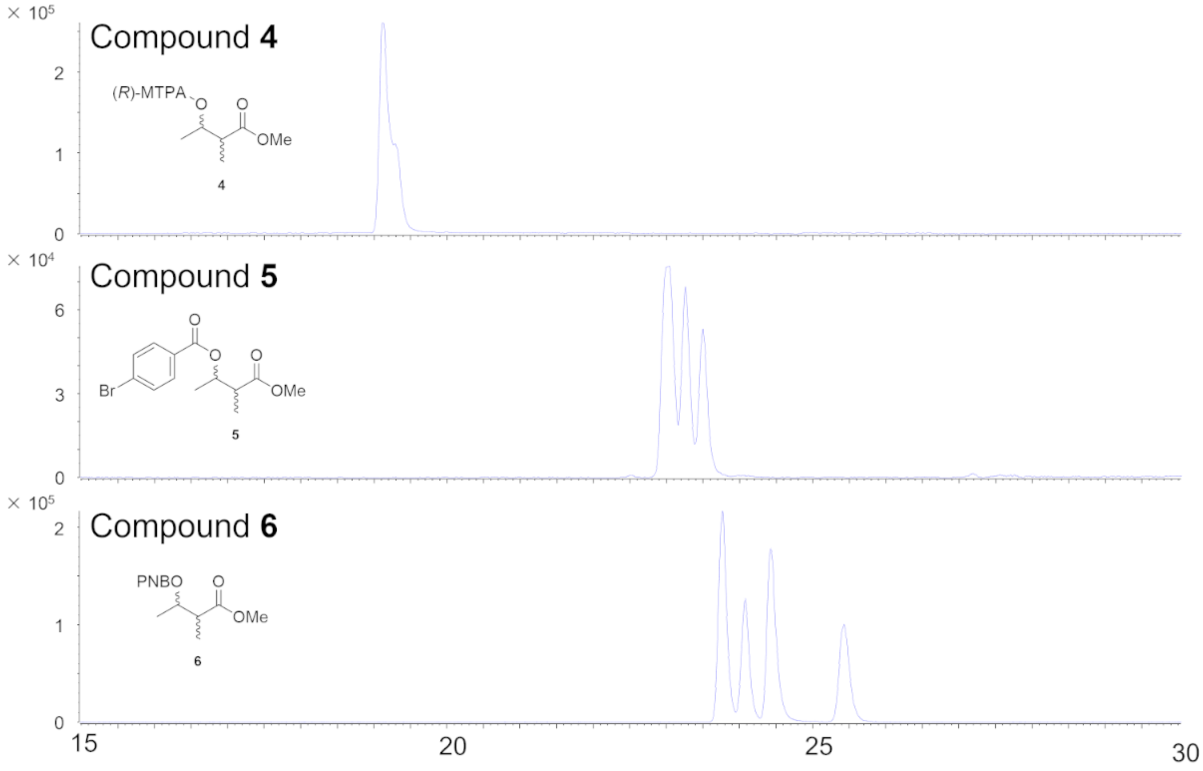

Figure S3. LC–MS chromatograms of ester **4**–**6** using CHIRALPAK ID-3 (MeOH conditions).

The enantiomeric and diastereomeric purity of 8–11.

Aliquots (5 μL) of each sample were injected into a CHIRALPAK ID-3 (4.6 × 250 mm, 3 μm) at a flow rate of 0.7 mL/min at 40 °C, with gradient elution from 50% MeOH to 100% MeOH, respectively. Gradient elution was performed using solvent A (H_2O) and solvent B (MeOH) with the following linear gradient combination: 50% (B) kept for 3 min, increased to 100% (B) over 23 min, and kept for 15 min. The enantiomeric and diastereomeric composition and HPLC chromatograms were shown as below.

Table S1. Enantiomeric and diastereomeric composition of synthetic standards **8**–**11** determined by chiral HPLC.

Compound	(2*S*,3*S*)-**8**<br>RT 27.0 min	(2*R*,3*S*)-**11**<br>RT 27.4 min	(2*R*,3*R*)-**10**<br>RT 27.7 min	(2S,3*R*)-**9**<br>RT 28.7 min
(2*S*,3*S*)-**8**	89.5%	8.1%	1.7%	0.6%
(2*R*,3*S*)-**11**	0.1%	96.1%	0.02%	3.7%
(2*R*,3*R*)-**10**	2.4%	0.1%	91.4%	6.1%
(2*S*,3R)-**9**	0.7%	0.3%	0.8%	98.2%

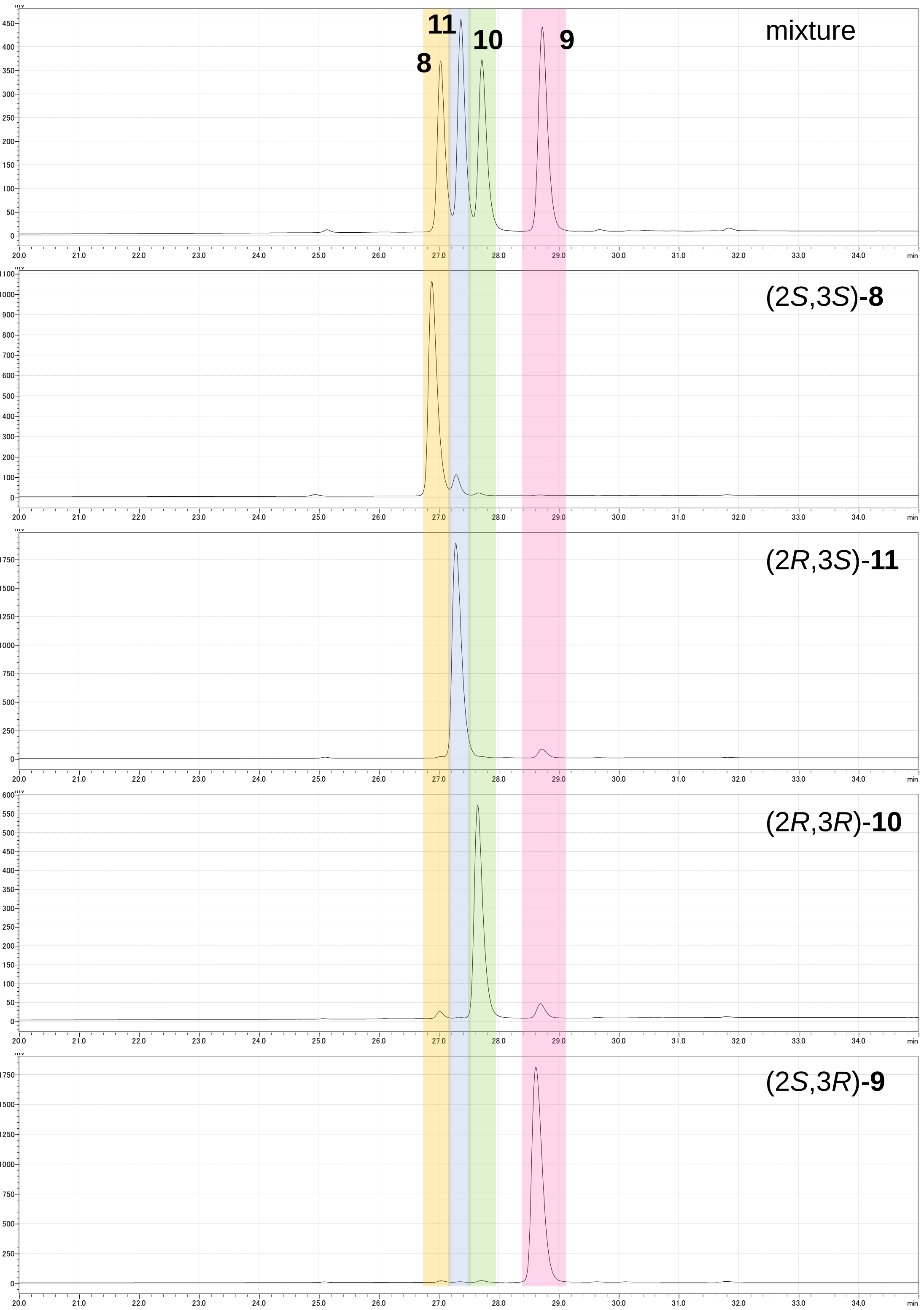

Figure S4. HPLC chromatograms of **8**–**11** using CHIRALPAK ID-3

3. NMR spectra

Table S2. ^{1}H NMR data (500 MHz) of capsulactone (1) in CD_3OD.
COSY correlations (boldline) and key HMBC correlations (arrow).

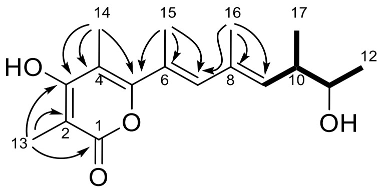

Position	δ_H, mult (*J* in Hz)	δ_C	COSY	HMBC
1		169.0		
2		98.2		
3		174.1[a]		
4		111.5		
5		160.0		
6		128.5		
7	6.05 (1H, brs)	139.2	H9, H15, H16	C5, C9, C15
8		132.8		
9	5.34 (1H, brd, *J* = 10.0 Hz)	136.7	H7, H10, H16	C7, C17
10	2.48 (1H, m)	41.9	H9, H11, H17	C11
11	3.54 (1H, dq, *J* = 6.4, 6,4 Hz)	72.7	H10, H12	
12	1.15 (3H, d, *J* = 6.4 Hz)	21.5	H11	C10, C11
13	1.88 (3H, s)	9.1		C1, C2, C3
14	1.97 (3H, s)	12.2		C3, C4, C5
15	2.03 (3H, d, *J* = 1.5 Hz)	16.8	H7	C5, C6, C7
16	1.85 (3H, d, *J* = 1.4 Hz)	17.1	H7, H9	C7, C8, C9
17	1.05 (3H, d, *J* = 6.7 Hz)	17.2	H10	C9, C10, C11

[a] assigned by HMBC data.

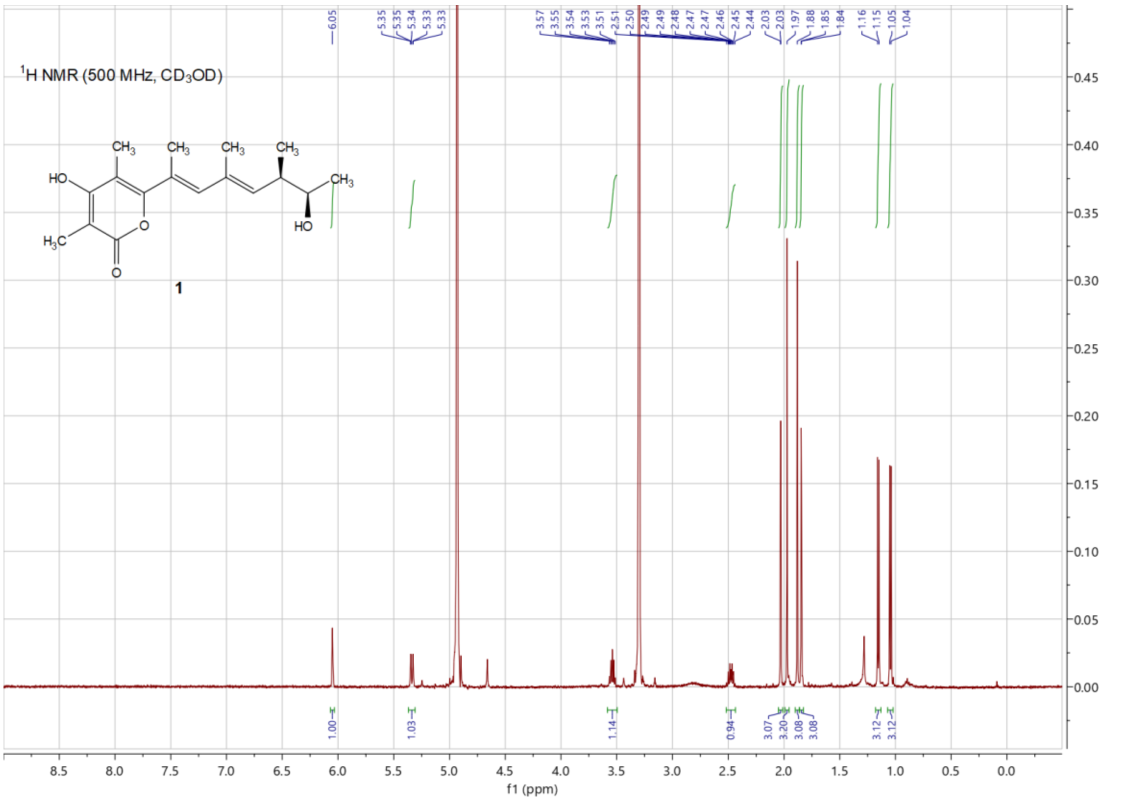

Figure S5. ^{1}H NMR spectrum of **1** in CD$_3$OD.

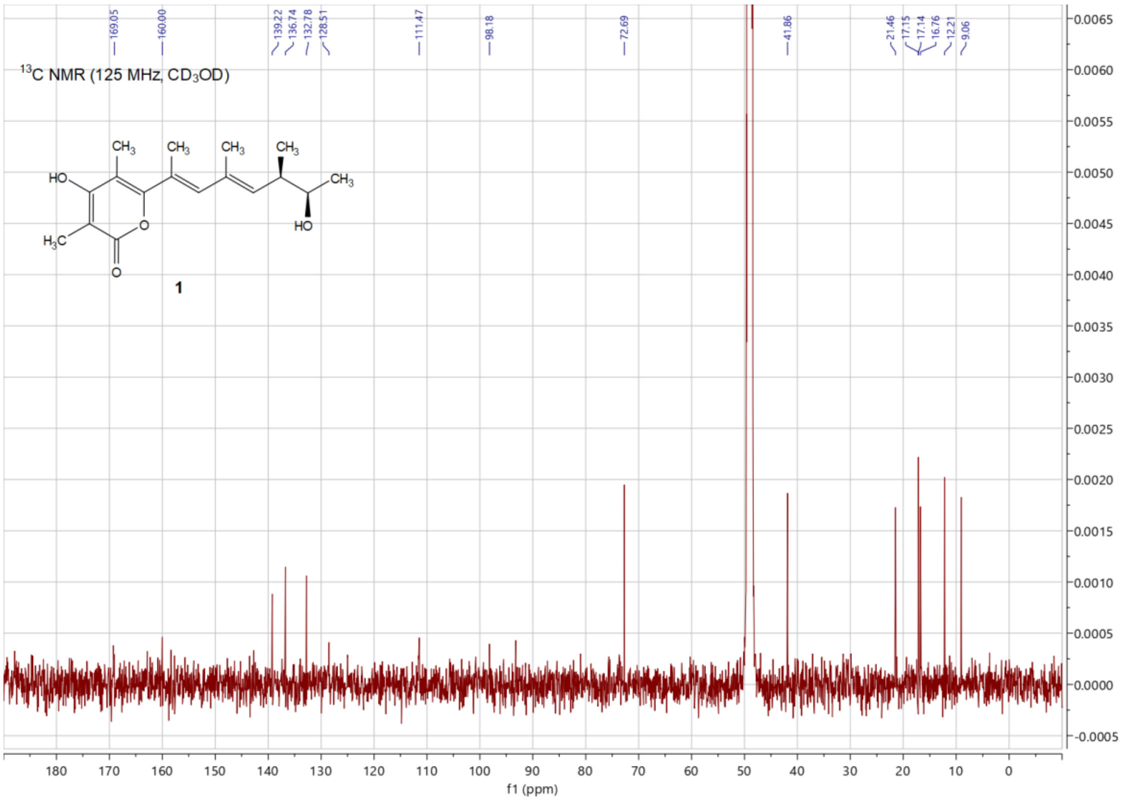

Figure S6. ^{13}C NMR spectrum of **1** in CD$_3$OD.

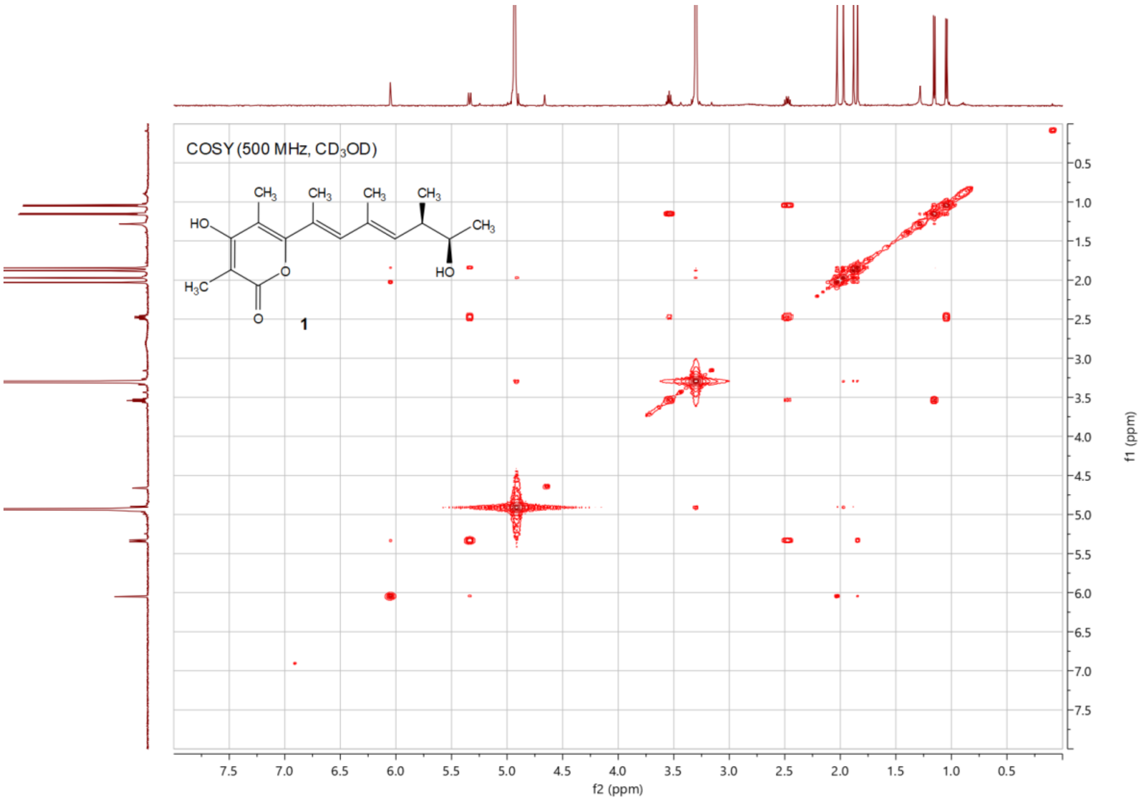

Figure S7. COSY spectrum of **1** in CD_3OD.

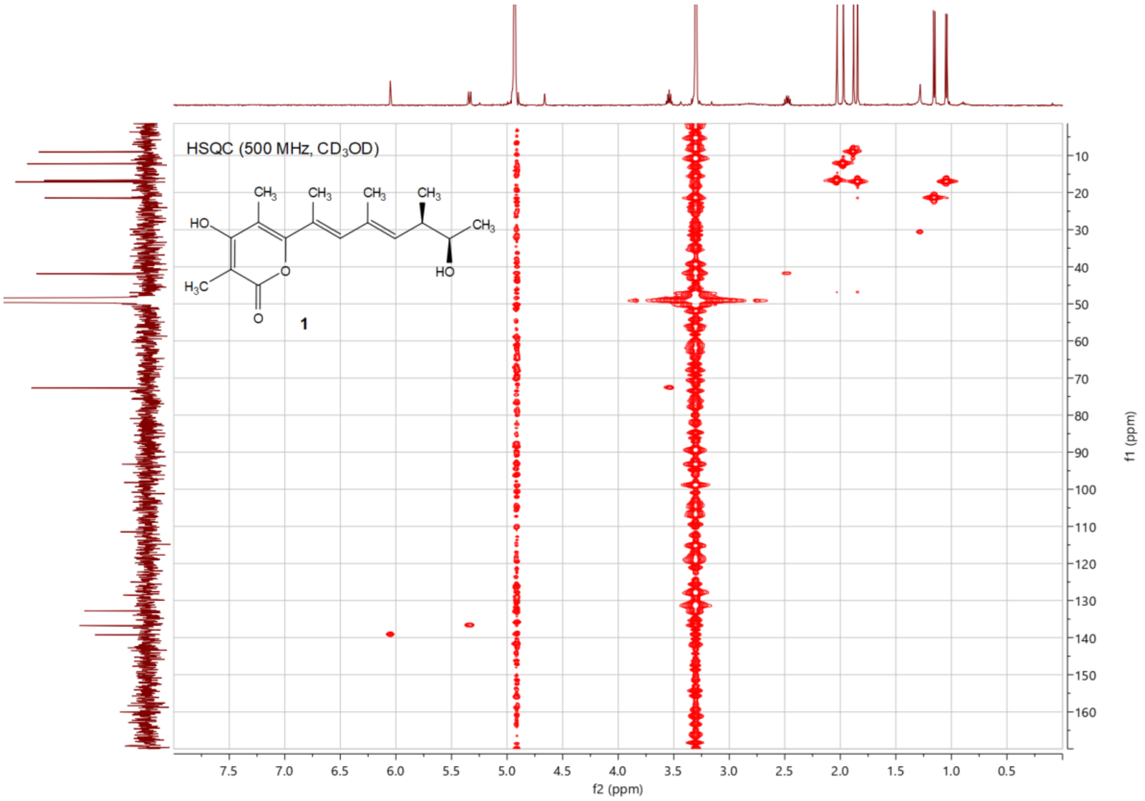

Figure S8. HSQC spectrum of **1** in CD_3OD.

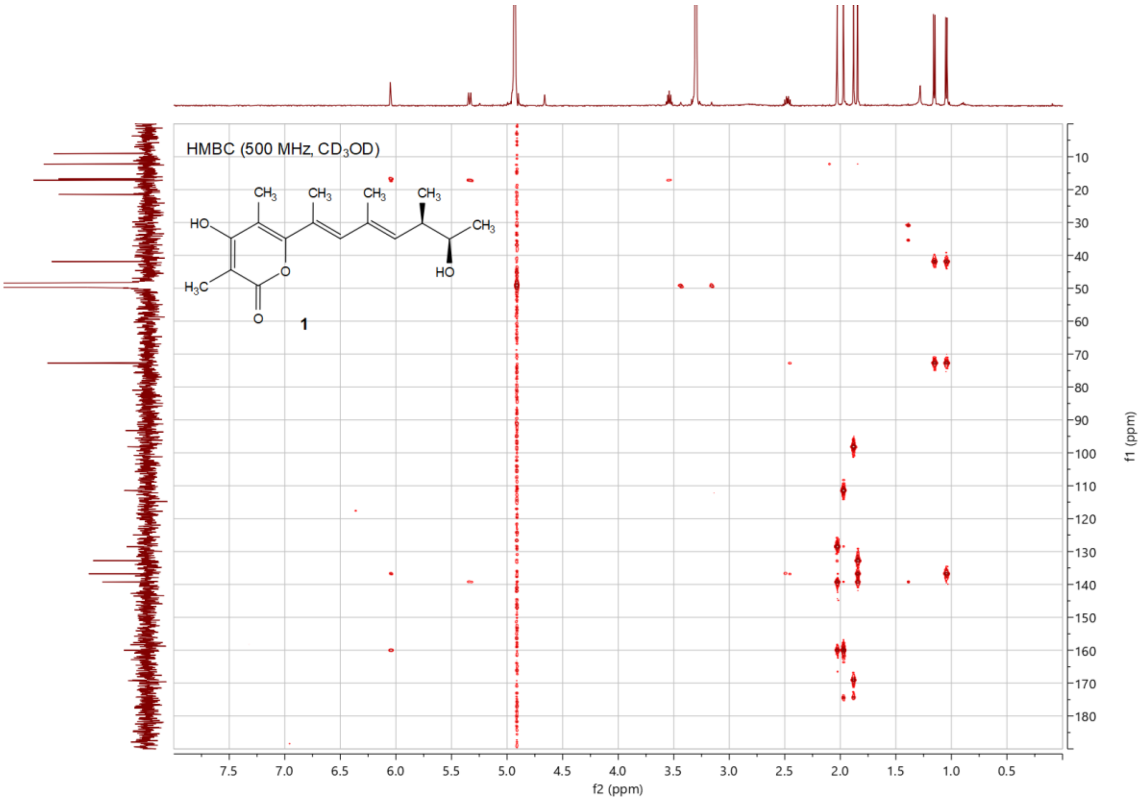

Figure S9. HMBC spectrum of **1** in CD_3OD.

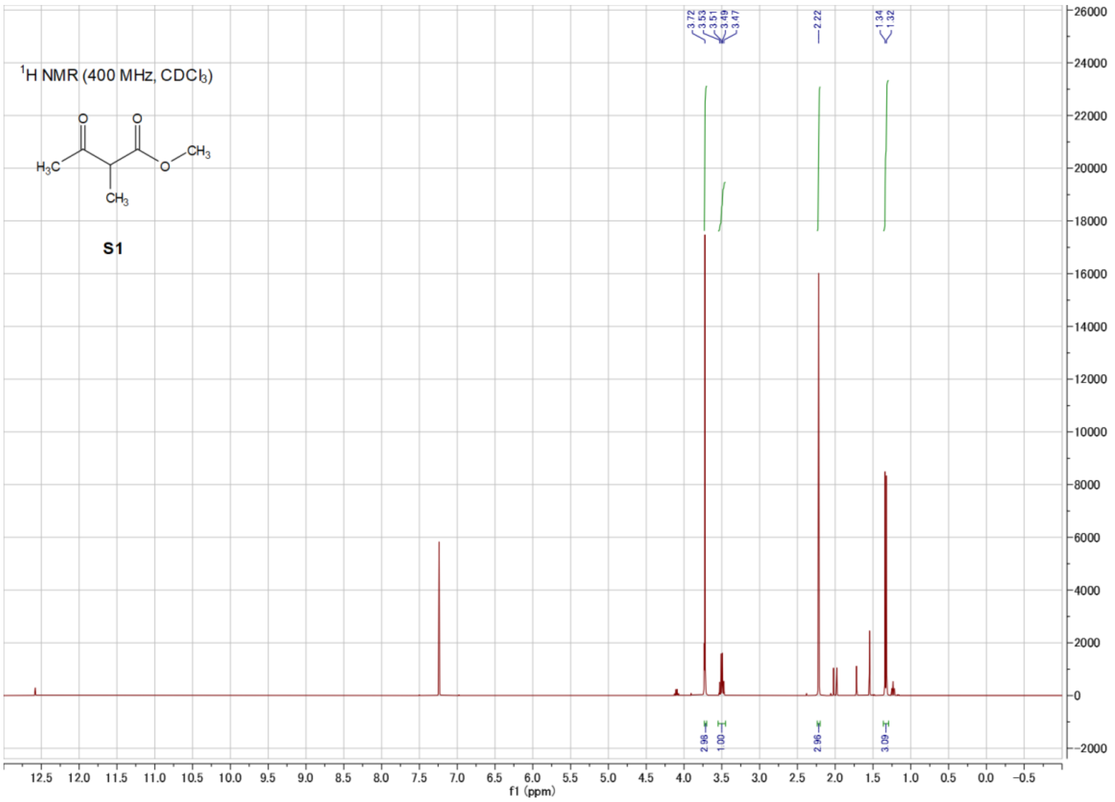

Figure S10. ^{1}H NMR spectrum of **S1** in $CDCl_3$.

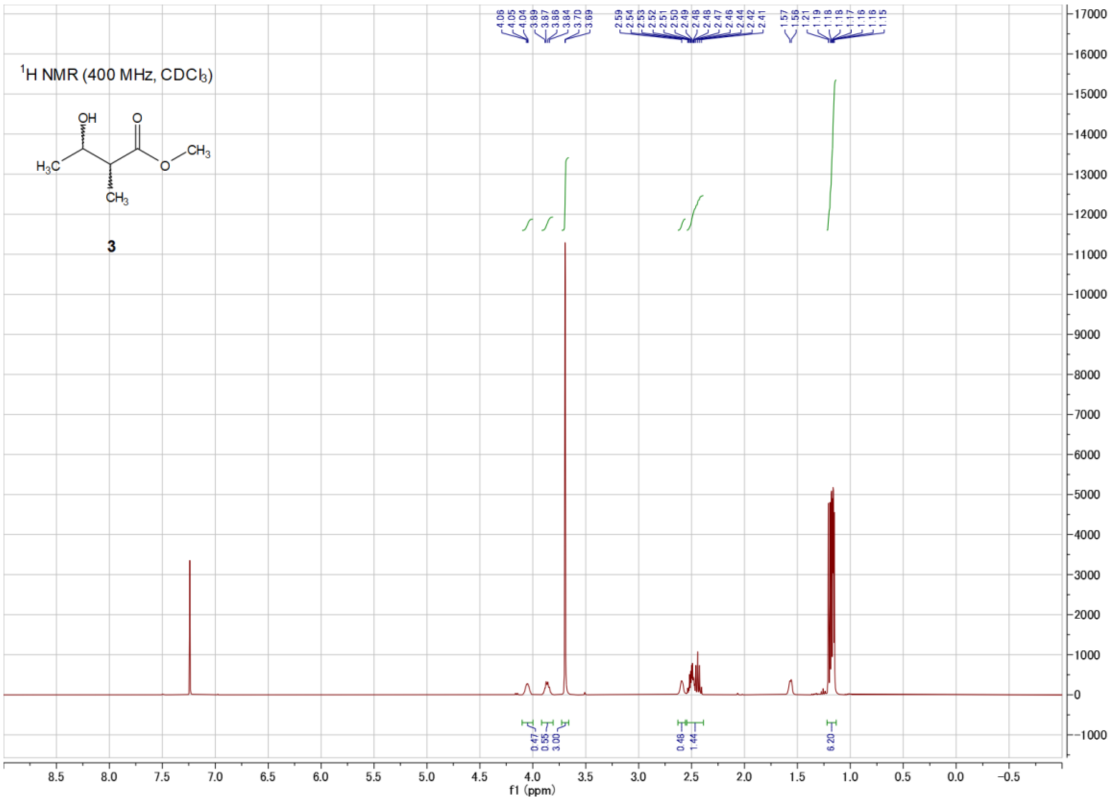

Figure S11. ^{1}H NMR spectrum of **3** in $CDCl_3$.

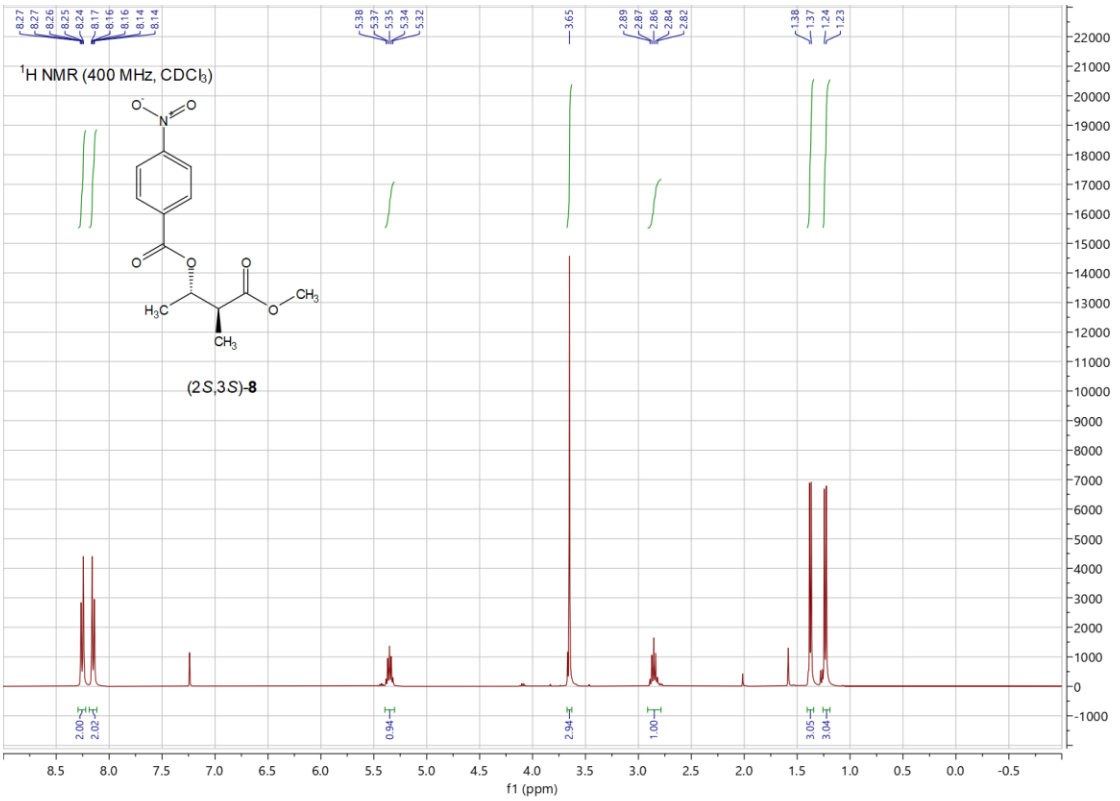

Figure S12. ^{1}H NMR spectrum of **8** in CDCl$_3$.

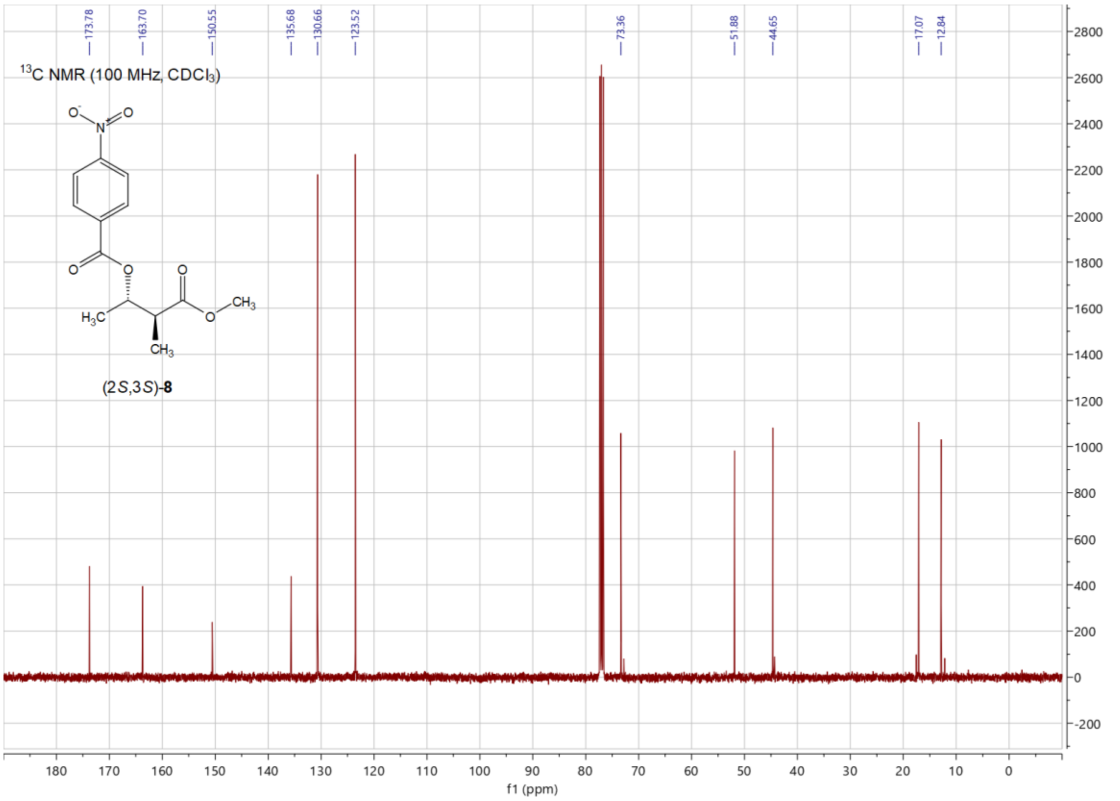

Figure S13. ^{13}C NMR spectrum of **8** in CDCl$_3$.

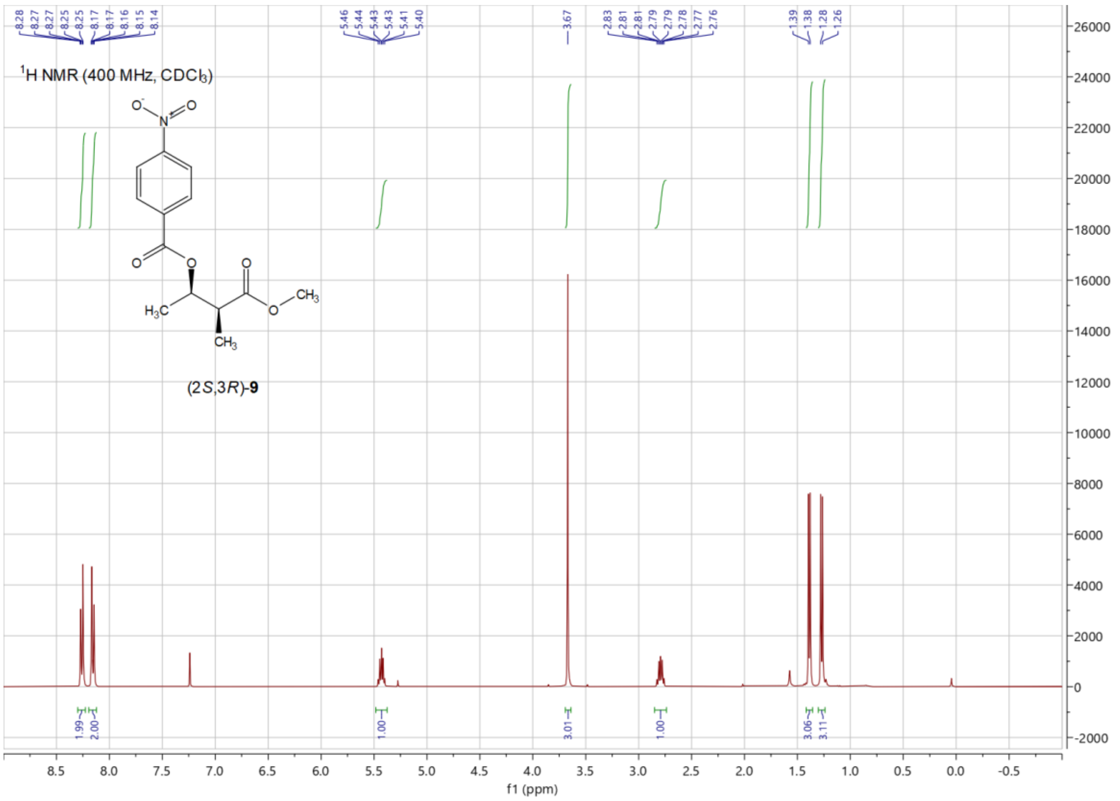

Figure S14. ^{1}H NMR spectrum of **9** in CDCl3.

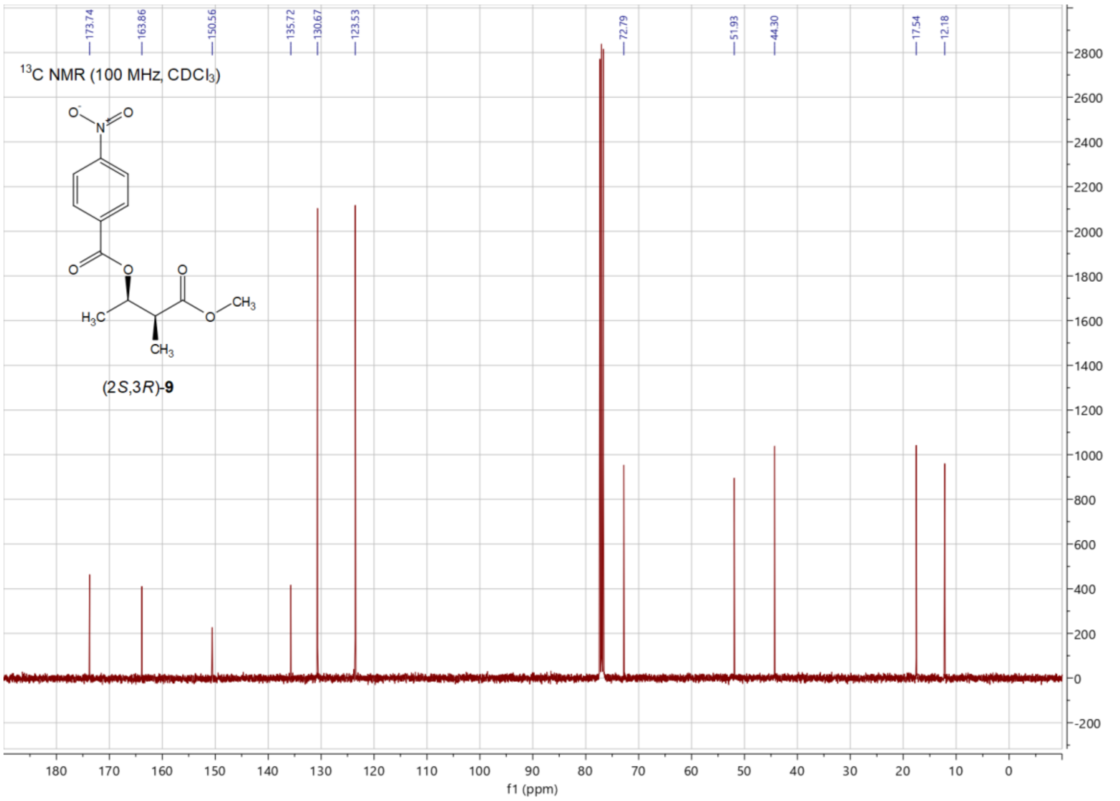

Figure S15. ^{13}C NMR spectrum of **9** in CDCl3.

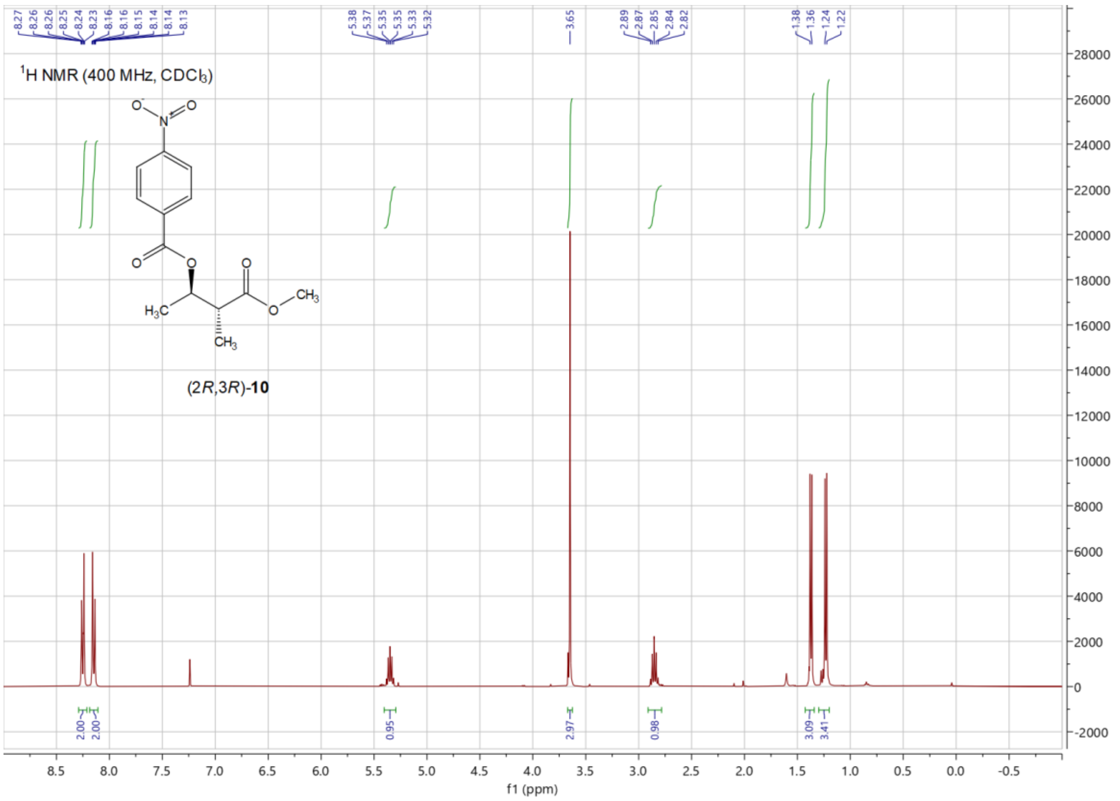

Figure S16. ^{1}H NMR spectrum of **10** in $CDCl_3$.

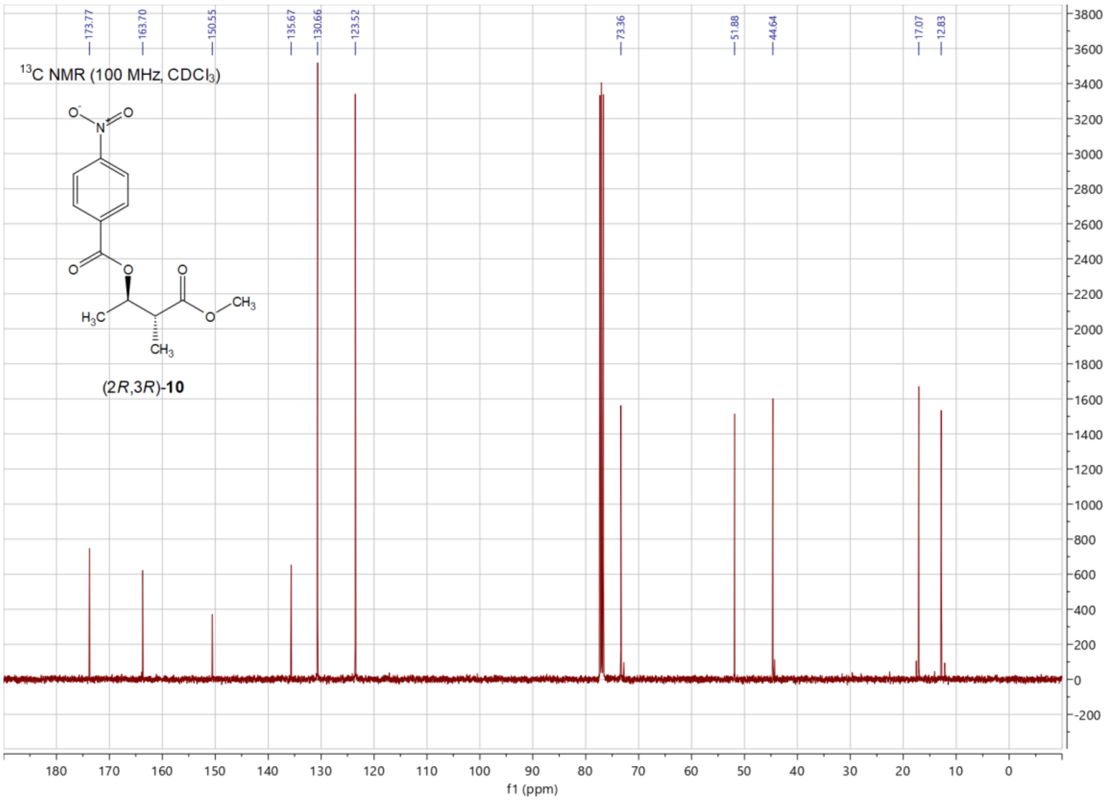

Figure S17. ^{13}C NMR spectrum of **10** in $CDCl_3$.

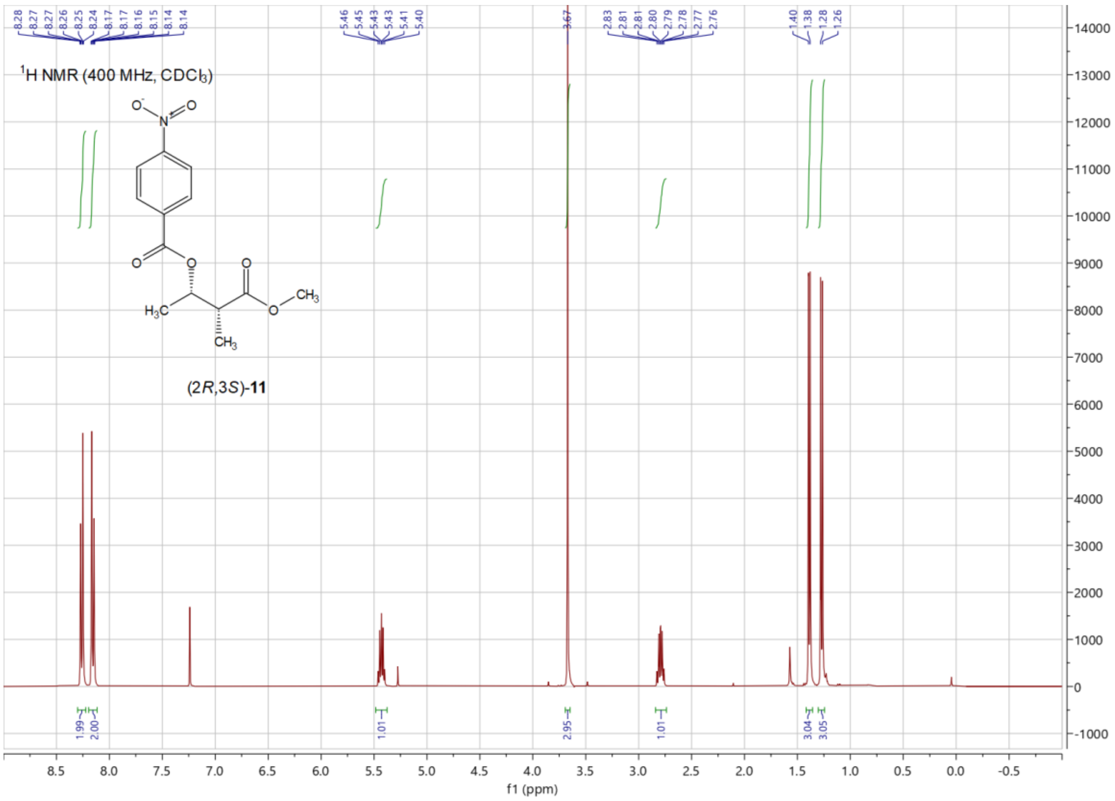

Figure S18. ^{1}H NMR spectrum of **11** in $CDCl_3$.

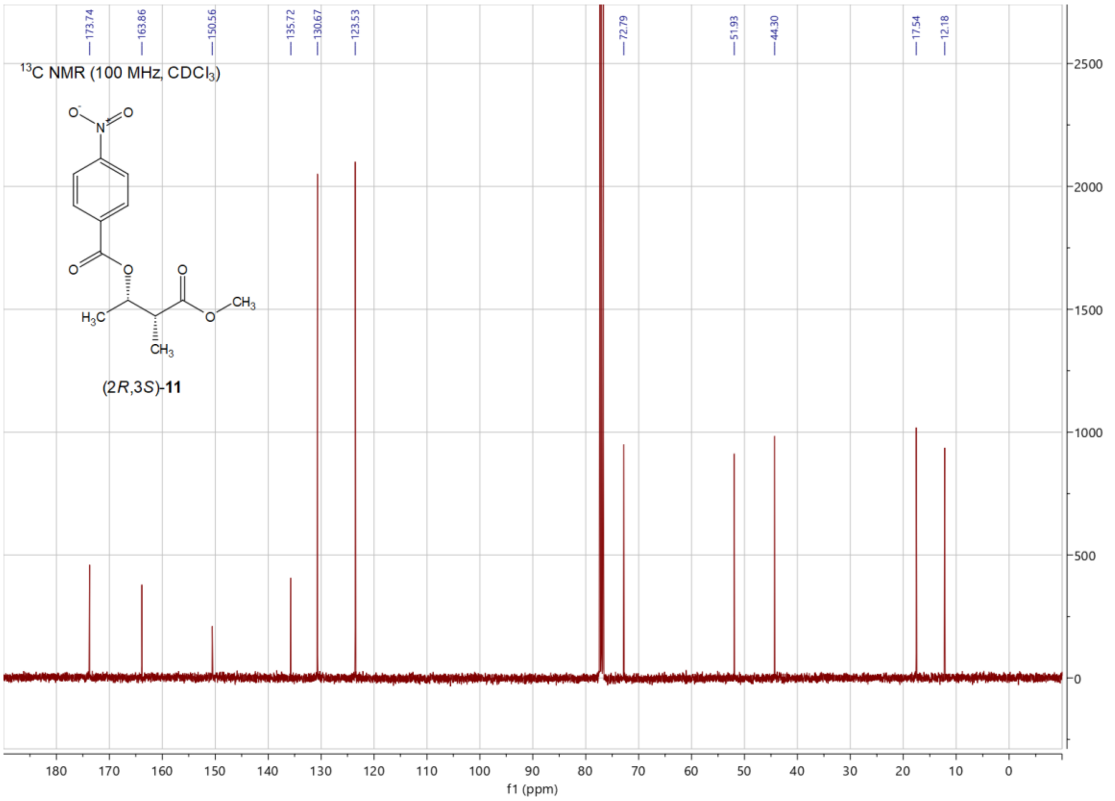

Figure S19. ^{13}C NMR spectrum of **11** in $CDCl_3$.
